# Immunopathology of childhood celiac disease—Key role of intestinal epithelial cells

**DOI:** 10.1371/journal.pone.0185025

**Published:** 2017-09-21

**Authors:** Grzegorz Pietz, Rituparna De, Maria Hedberg, Veronika Sjöberg, Olof Sandström, Olle Hernell, Sten Hammarström, Marie-Louise Hammarström

**Affiliations:** 1 Department of Clinical Microbiology, Immunology, Umeå University, Umeå, Sweden; 2 Department of Clinical Sciences, Pediatrics, Umeå University, Umeå, Sweden; Tulane University, UNITED STATES

## Abstract

**Background & Aims:**

Celiac disease is a chronic inflammatory disease of the small intestine mucosa due to permanent intolerance to dietary gluten. The aim was to elucidate the role of small intestinal epithelial cells in the immunopathology of celiac disease in particular the influence of celiac disease-associated bacteria.

**Methods:**

Duodenal biopsies were collected from children with active celiac disease, treated celiac disease, and clinical controls. Intestinal epithelial cells were purified and analyzed for gene expression changes at the mRNA and protein levels. Two *in vitro* models for human intestinal epithelium, small intestinal enteroids and polarized tight monolayers, were utilized to assess how interferon-γ, interleukin-17A, celiac disease-associated bacteria and gluten influence intestinal epithelial cells.

**Results:**

More than 25 defense-related genes, including IRF1, SPINK4, ITLN1, OAS2, CIITA, HLA-DMB, HLA-DOB, PSMB9, TAP1, BTN3A1, and CX3CL1, were significantly upregulated in intestinal epithelial cells at active celiac disease. Of these genes, 70% were upregulated by interferon-γ via the IRF1 pathway. Most interestingly, IRF1 was also upregulated by celiac disease-associated bacteria. The NLRP6/8 inflammasome yielding CASP1 and biologically active interleukin-18, which induces interferon-γ in intraepithelial lymphocytes, was expressed in intestinal epithelial cells.

**Conclusion:**

A key factor in the epithelial reaction in celiac disease appears to be over-expression of IRF1 that could be inherent and/or due to presence of undesirable microbes that act directly on IRF1. Dual activation of IRF1 and IRF1-regulated genes, both directly and via the interleukin-18 dependent inflammasome would drastically enhance the inflammatory response and lead to the pathological situation seen in active celiac disease.

## Introduction

Celiac disease (CD) is a chronic small intestinal immune-mediated enteropathy caused by permanent intolerance to the food antigen gliadin in wheat gluten and related prolamines in barley and rye [1,2]. CD is a multifactorial disease with a strong genetic association to the major histocompatibility complex (MHC) class II alleles for HLA-DQ2 and/or HLA-DQ8 and carrying at least one of these is a prerequisite for contraction of the disease [1]. In CD patients, intake of gluten causes an inflammatory lesion in the small intestine, characterized by villous atrophy and crypt hyperplasia and increased numbers of T lymphocytes both within the epithelium, so called intraepithelial lymphocytes (IELs), and the lamina propria. Immunological manifestations involve production of IgA antibodies to gliadin and the autoantigens tissue transglutaminase-2 (tTG) and endomysium as well as production of the pro-inflammatory cytokines interferon (IFN)-γ, interleukin (IL)-17A and IL-21 and the down-regulatory cytokines IL-10 and transforming growth factor (TGF)-β1 [3–9]. Clinical and histological improvement, as well as normalization of autoantibody titers and cytokine production is seen upon withdrawal of gluten from the diet and life-long, strict gluten-free diet is today the only treatment [10,11].

T lymphocytes play a central role in the pathogenesis of CD and gluten specific CD4 and CD8 expressing T lymphocytes have been isolated from the small intestinal mucosa of CD patients [12,13]. The gluten specific CD4^+^T helper cells are mainly located in the lamina propria [4]. However, there are indications that an epithelial reaction plays a central role in initiating and maintaining CD, i.e. CD8^+^IELs constitute the major cellular source of IFN-γ, IL-17A, and IL-10 in the inflamed intestinal mucosa of biopsies collected at diagnosis [6,8,14]. An important role for IELs in CD is underscored by the demonstration of over-stimulated cytotoxic CD8^+^IELs that have lost their antigen restriction in active disease giving the reaction an innate trait [15,16]. Locally produced IL-15 has been suggested to have a pivotal role in supporting development of this cytolytic capacity in IELs [16,17]. The epithelium is attacked from the apical side of the intestinal epithelial cells (IECs) by dietary gluten but also by components of the microbiota. Notably, we have previously demonstrated that there are bacteria adherent to the epithelium that are over-represented in CD patients, among these two new species that were isolated from small intestinal biopsies of CD patients [18–21], and that an IL-17A response is seen when biopsies of CD patients with inactive disease are challenged *ex vivo* with these CD-associated bacteria [8].

In active CD, most of the key cytokine IFN-γ is produced by IELs [6,14], yet little is known about their neighboring cells, the IECs. To elucidate the role of IECs in the immune pathology of CD we analyzed gene expression differences in purified IECs from duodenal mucosa of CD patients with active disease, CD patients with inactive disease and clinical controls. Two *in vitro* models for human intestinal epithelium, enteroids and polarized tight monolayers, were utilized in order to assess how IFN-γ and IL-17A, two cytokines secreted by IELs in active CD, might influence IEC function. Further, challenge of tight monolayers with CD-associated bacteria in the presence or absence of gluten peptides was performed in order to determine to what extent these bacteria contribute to alterations in IEC function. The results suggest a clear immunological function of IECs in CD. Thus, significantly elevated levels of the protease inhibitor SPINK4 and the lectin ITLN1 suggest innate immunity to bacteria, elevated levels of OAS2 suggest innate immunity to virus, elevated levels of CIITA, HLA-DMB and HLA-DOB together with high levels of MHC class II molecules, particularly HLA-DR and HLA-DQ, suggest ample antigen presentation to T helper cells but with altered antigen repertoire displayed, elevated levels of PSMB9 and TAP1 suggest induction of a functional immunoproteasome with changes in display of T-cell epitopes on MHC class I molecules as is seen in immune responses to viral infections, increased levels of butyrophilin/BTN3A1 suggest presentation of bacterial antigens to γδT-cells and elevated levels of the chemokine CX3CL1 and accumulation of secreted CX3CL1 at the basal lamina indicates recruitment of dendritic cells to the vicinity of the epithelium and possibility of increased antigen sampling from the gut lumen. Upregulation of IRF1 by IFN-γ and/or CD-associated bacteria seems to be a key factor for induction of the majority of these genes.

## Materials and methods

### Patients

Patients were enrolled on a continuous basis and belonged to one of three diagnostic groups; Active CD: 7 boys and 19 girls [median (interquartile range (IQR)): 11.5 (7.1–13.0) years] showing serum anti-tTG IgA levels ≥5 U/mL and duodenal mucosa with histological changes classified as Marsh score 3a–c. Treated CD: 1 boy and 4 girls [14.5 (14.2–14.6) years] with anti-tTG IgA levels between 1.6 and 18.4 U/mL and Marsh score 0–1, who had been on a gluten-free diet for >5 months. Clinical controls: 9 boys and 16 girls [10.2 (5.2–16.5) years] with no known food intolerance, anti-tTG IgA levels <5 U/mL and Marsh score 0 (S1 Table).

### Biopsies

Duodenal biopsies were collected by upper endoscopic examination from children admitted to the Department of Pediatrics, Umeå University Hospital for routine examination or clinical follow-up. Biopsies were taken by endoscopic procedure. Four biopsies were placed in formaldehyde and used for clinical routine histopathological diagnostics. Three to six biopsies were immediately placed in ice-chilled HEPES-buffered RPMI1640 and was used for isolation of IECs, isolation of CD3^+^IELs, isolation of crypts for enteroid culture, or frozen for immunohistochemistry and immunofluorescence.

### Isolation of IECs

IECs were isolated by positive selection from cell suspensions of fresh duodenal biopsies using a slightly modified procedure of that previously described [18]. The biopsies were washed with phosphate buffered saline (PBS; pH 7.2), incubated with 0.1 mM dithiotreitol (DTT) in Medium 199 under vigorous shaking at room temperature for 20 min and then retrieved by centrifugation at 1500 × g for 5 min. Pelleted cells and tissue pieces were subsequently treated with 72.5 units (U)/mL collagenase type IV (Worthington, Freehold, NJ) in heat inactivated human AB^+^ serum under vigorous shaking at 37°C for 40 min. The cell suspension was then passed through a stainless steel sieve and washed twice with RPMI1640. Thereafter the cell suspension was depleted of leucocytes and sticky cells by treatment with paramagnetic beads charged with anti-CD45 monoclonal antibodies (mAbs) (Dynabeads CD45 Cell kit, Invitrogen Dynal Biotech, Oslo, Norway). Beads were added at a bead to cell ratio of 20:1 and incubated at 4°C for 30 min with slow end-over-end rotation. Cells bound to the beads were retrieved by treatment with a magnet and discarded. Unbound cells were collected and residual bead-bound cells removed from the suspension of unbound cells by two additional treatments with magnet. Thereafter, IECs were collected from the suspension of unbound cells by positive selection using magnetic beads charged with BerEP4, a mAb that is specific for an antigen expressed on the cell surface of IECs (CELLection Epithelial Enrich system, Invitrogen Dynal Biotech) [22]. Beads were added at a ratio of 20:1 and incubated with the cell suspension at 4°C for 30 min with slow end-over-end rotation and then beads with bound cells were collected by treatment with a magnet. The beads with bound IECs were washed twice with RNase-free PBS, frozen in RTL-buffer (RNeasy Mini Kit; Qiagen, Sollentuna, Sweden) supplemented with 0.1 M 2-mercaptoetanol and stored at -80°C until RNA extraction. Cell yields were estimated from amounts of 18S rRNA extracted. The content of 18S rRNA in extracted RNA samples was determined by real-time quantitative reverse transcriptase-polymerase chain reaction (qRT-PCR), as described below. Previous analysis of over 100 human IEC samples showed that the amount of 18S rRNA per IEC is approximately 1 U and seemingly constant for IECs from small and large bowel of normal and inflamed mucosa [23]. Median total amounts of 18S rRNA retrieved were 105,419 (IQR = 29,458–324,040) U for IECs of CD patients with active disease (n = 14), 812,744 (IQR = 539,641–1.2 x10^6^) U for IECs of CD patients with treated CD (n = 5), and 129,612 (IQR = 84,199–257,448) U for IEC of clinical controls (n = 9). Hybridization bead array analysis of IEC samples showed that contamination of IECs by leukocytes was very low (S1 File). T cells were the main contaminants. Still the signals of T cell markers (CD2, CD3D, CD3E, CD3G, CD4, CD5, CD8, CD247) were all below 10% of the respective signal in CD3^+^IELs (S1 File). All markers of B cells, plasma cells, NK cells, monocytes/macrophages/dendritic cells and granulocytes, except CD14 (158 arbitrary units (AU)) and CD40 (83 AU), were negligible with signals below 20 AU.

### Isolation of CD3^+^IELs

Intestinal CD3^+^IELs were isolated from fresh duodenal biopsies as previously described with slight modifications [6]. The biopsies were first washed with PBS and then incubated with 0.1 mM DTT in Medium 199 with 10% heat inactivated human AB^+^ serum at room temperature for 20 min under vigorous shaking and retrieved by centrifugation 1500 × g for 5 min. Pelleted cells and remaining biopsy pieces were re-suspended and vortexed twice for 4 minutes. Cells in the supernatant were subjected for positive selection by paramagnetic beads charged with anti-CD3 mAb (Dynabeads CD3, Invitrogen Dynal Biotech) for 30 min at 4°C under slow end-over-end rotation. Bound cells (CD3^+^IELs) were collected by magnet, washed in RNase-free PBS and frozen in RTL-buffer (RNeasy Mini Kit; Qiagen) supplemented with 0.1 M 2-mercaptoetanol and stored at -80°C until RNA extraction.

### Isolation of crypts, establishment of human enteroid cultures and cytokine stimulation conditions

Enteroid cultures were established from crypts isolated of duodenal biopsies according to Sato et al [24]. Biopsies were collected in 5 mL base medium comprising advanced Dulbecco’s modified Eagle medium (Invitrogen, Paisley, UK) supplemented with 10 mM HEPES buffer (pH 7.4), 0.2 mM GlutaMAX (Gibco, Grand island, NY) and 100 U/mL penicillin and 85μg/mL streptomycin. The biopsies were washed five times with PBS and then incubated in PBS containing 2 mM EDTA at 4°C for 30 min to release crypts. The number of crypts were counted under the microscope and centrifuged at 1200 rpm for 5 min to isolate the crypts as a pellet. The pellet was suspended in a mixture consisting of 80% Matrigel (BD Bioscience, Stockholm, Sweden) and 20% complete medium composed of 30% volume/volume base medium supplemented with 50% volume/volume Wnt3a-conditioned medium, 20% volume/volume R-spondin 1-conditioned medium, 1x B27 supplement (Thermo Fisher Scientific, Gibco), 0.1% human recombinant noggin (catalogue number H6416, Sigma-Aldrich, St. Louis, MO), 1 mM N-acetylcysteine (Sigma-Aldrich), 50 ng/mL human recombinant epidermal growth factor, 1 μg/mL [Leu-15] gastrin (catalogue number G9145 Sigma-Aldrich), 10 mM nicotinamide (Sigma-Aldrich), 500 nM A83-01 (chemical name: 3-(6-Methyl-2-pyridinyl)-*N*-phenyl-4-(4-quinolinyl)-1*H*-pyrazole-1-carbothioamide), (Tocris Bioscience, R&D Systems, Minneapolis, MN; catalogue no. 2939), 10 mM SB202190 (p38 MAPK inhibitor) (Sigma-Aldrich). In this way a suspension of crypts in Matrigel and complete medium was made and 50 μL of this suspension was added to each well of a 24 well tissue-culture plate (Corning, Corning, NY) so that each well contained 10–50 crypts in 50 μL Matrigel-complete medium mixture. To each well 500 μL complete medium was added. Crypts were grown in Matrigel supported by complete medium for ten days at 37°C in humid atmosphere with 5% CO_2_. The medium was replaced with fresh medium every third day.

For cytokine treatment of enteroids the medium of quadruplicate ten day old enteroid cultures in separate wells was replaced by complete medium supplemented 200 U/mL recombinant IFN-γ (Life Technologies, Carlsbad, CA; catalog no. PHC4031) or 100 ng/mL recombinant IL-17A (e-biosciences; catalog no. 34-8179-82) and thereafter incubated for 24 h at 37°C in 5% CO_2_. Parallel quadruplicate cultures enteroid cultures given fresh complete medium only served as experimental control and provided the basis for calculations of relative quantity (RQ). After incubation the enteroids were washed in RNase-free PBS and frozen in RTL-buffer (RNeasy Mini Kit) supplemented with 0.1 M 2-mercaptoetanol and stored at -80°C until RNA extraction. For characterization of enteroids and confirming the presence of pleuripotent stem cells, analysis of mRNA for the R-spondin receptor LGR4 was performed using real-time quantitative RT-PCR. Only results from experiments in which STAT1 and/or CIITA mRNA levels showed statistically significant increase after IFN-γ stimulation and statistically significant increase in CCL20 mRNA levels after IL-17A stimulation are included.

### CD associated bacteria

Eight bacterial isolates from proximal jejunum of two CD patients (*Prevotella jejuni* isolates CD3:27, CD3:28, and CD3:33, *P*. *histicola* isolate CD3:32, *P*. *melaninogenica* isolate CD3:34, *Lachnoanaerobaculum umeaense* isolate CD3:22, and *A*. *graevenitzii* isolate CD4:Bx9) [19–21] were cultivated under anaerobic conditions (5% H_2_ and 10% CO_2_ in N_2_) on blood agar plates (Colombia Blood Agar Base; Acumedia, Neogene, Ayr, UK) supplemented with 5% defibrinated horse blood. Colonies were picked and washed in PBS and the density was adjusted to 4 on the McFarland optical scale, representing 1.2 x 10^9^ bacteria/mL and thereafter suspended in challenge medium (one part RPMI 1640 containing 0.4% human serum albumin and one part Medium199 containing 15% human normal AB^+^ serum, 2 mM Na-pyruvate and a supplement of non-essential amino acids) to a concentration of 1 x 10^8^ bacteria/mL. For challenge with bacteria mix 1:1, each bacterial isolate was suspended in 1 mL of challenge medium, and thereafter 100 μL of each bacterial isolate suspension was added together yielding a final concentration of 1 x 10^8^ bacteria/mL for each isolate.

### Establishment of human intestinal epithelial cell polarized tight monolayers and challenge conditions

The colon carcinoma cell line T84 (American Type Culture Collection, Rockville, MD) was cultured at 37°C in a humidified atmosphere with 5% CO_2_. Tissue culture medium used was a 1:1 mixture of Dulbecco's modified Eagle's medium and Ham’s F12 medium supplemented with 15 mM HEPES buffer (pH 7.4), 8% fetal calf serum, 2 mM L-glutamine, 100 U/mL penicillin and 85μg/mL streptomycin. All tissue culture media components were from Invitrogen. Confluent cultures were trypsinized by incubation for 5 min at 37°C with 0.25% trypsin and 0.5% EDTA in PBS (pH 7.2). After trypsinization cells were allowed to recover for 60 min at 37°C in complete medium.

Tight monolayers were established by seeding 0.5×10^6^ T84 cells in 0.5 mL complete culture medium per well in transwell inserts with semi-permeable polycarbonate membrane supports with 12 mm diameter and 0.4 μm pore size (Costar 3401; Corning). Complete culture medium (1.5 mL) was also added to the outside of the inserts. From the second day after seeding, medium was changed every day until a confluent monolayer with a transepithelial electrical resistance of ≥ 1000 Ohm/cm^2^ was obtained [25]. Transepithelial electrical resistance was measured by using the Millicell Electrical Resistance System (Millipore, Bedford, MA) with chopstick electrodes.

Stimulation with IFN-γ and IL-17A was done from the basolateral side by replacing the culture medium in the outer chamber with 1.5 mL culture medium containing 200 U/mL of recombinant human IFN-γ (Life Technologies) or 50 ng/mL of recombinant human protein carrier-free IL-17A (e-biosciences), followed by incubation for 4 h, 24 h and 72 h at 37°C in an humidified atmosphere with 5% CO_2_.

Stimulation with CD-associated bacteria and gluten was done from the apical side by replacing the media in the upper chamber with 0.5 mL challenge medium containing 5x10^7^ bacteria with or without 1 mg trypsin-treated gluten/mL [8,26]. For each stimulant three to four parallel wells were used to carry out the experiment and wells treated with challenge medium only served as experimental controls. The transwell plates with tight monolayers were placed in an anaerobic gas chamber (Anoxomat, Mart Microbiology, Norwood, MA) from which air was pumped out and replaced with 5% H_2_ and 10% CO_2_ in N_2_. The anaerobic gas chamber with the tight monolayers was placed in an incubation chamber with a constant temperature of 37°C for four hours.

At the end of incubation the membranes with tight monolayers were cut out, washed two times with RNase-free PBS and frozen in RTL-buffer (RNeasy Mini Kit; Qiagen) supplemented with 0.1 M 2-mercaptoetanol and stored at -80°C until RNA extraction. Sham-treated parallel tight monolayer cultures constituted the negative controls and provided the basis for calculations of relative quantity (RQ).

### Gene expression analysis at the mRNA level

#### RNA extraction

Total RNA was extracted from IECs, CD3^+^IELs, and challenged and sham-treated enteroids and tight monolayers by using the RNeasy Mini Kit (Qiagen) according to the manufacturer's instructions and dissolved in RNase-free water containing rRNasin ribonuclease inhibitor (Promega, Madison, WI) and stored at -80°C until gene expression analysis by genome-wide hybridization bead array screening, real-time quantitative polymerase chain reaction array (qPCR-array) and/or qRT-PCR. The concentration of 18S rRNA was determined in each sample using real-time qRT-PCR (Applied Biosystems) in which triplicates of the samples were compared to triplicates of serial dilutions of a pool of total RNA extracted from polyclonally stimulated peripheral mononuclear cells [27]. Results are expressed as U/μL where one U was defined as the amount in 10 pg of the RNA standard.

#### Gene expression analysis using genome-wide hybridization bead array screening of cRNA libraries

Four samples of purified IECs from duodenal mucosa of CD patients with active disease, 4 samples of purified IECs of clinical controls, 5 samples of purified CD3^+^IELs from CD patients with active disease and 3 samples of purified CD3^+^IELs of clinical controls as well as T84 polarized tight monolayers challenged with bacteria or sham-treated were individually subjected to genome-wide hybridization bead array analysis for gene expression. Concentrations and purity of total RNA samples were determined by measuring optical density at 260 nm (OD260) and OD280 in a NanoDrop 1000 spectrophotometer V3.0.0 (Saveen Werner, Limhamn Sweden). Four hundred ng of total RNA with OD260/OD280 ratios ≥1.8, was converted to biotinylated double stranded cRNA by *in vitro* transcription and amplification according to the protocol of the Illumina Totalprep RNA Amplification Kit (Ambion, Austin, TX). The procedure yielded > 15 μg cRNA and the purity estimated as OD260/OD280 was ≥1.8. Agarose gel analysis of sample integrity in a 2100 Bioanalyser (Agilent Technologies, Palo Alto, CA) showed cRNAs suitable for hybridization with normal distribution of fragments between 200 and 6,000 base pairs in length. The labeled cRNA samples were then hybridized individually to Sentrix HumanRef-8_v3 expression BeadChip (18,401 genes; IEC and CD3^+^IEL samples) or HumanHT-12 v4 BeadChip (34,602 genes; T84 tight monolayer samples) incubated with streptavidin-Cy3 and scanned on the Illumina BeadStation GX (both types of BeadChip and the BeadStation were from Illumina, San Diego, CA).

The raw data was analyzed using Illumina Beadstudio software (version 3.1). Background was first subtracted from the data and then normalized using Beadstudios cubic spline algorithm. Significant differential expression was calculated using Beadstudio software by applying Illumina Custom algorithm. Raw and analyzed data-files from hybridization bead array were submitted to Gene Expression Omnibus (GEO) DataSets (www.ncbi.nlm.nih.gov/geo) with accession numbers GSE102991 for IEC samples, GSE102993 for CD3^+^IEL samples and GSE103100, GSE103107, and GSE103374 for T84 tight monolayer samples. To avoid selecting genes with high fold change due to low signal intensity a minimum signal intensity value of ≥ 15 AU in the group of CD patients with active disease. Signals of controls that gave negative values were assigned a value of 0. When medians of controls became 0 they were assigned a value of 0.01. Relative quantity (RQ) was calculated as the median signal value of CD patient group through the median signal value of the control group. The analysis was focused on upregulated genes. Therefore potentially down-regulated genes were excluded and only genes with an RQ ≥ 1 were included in data analyses performed to find appropriate cut-offs for RQ and signal strength.

#### Gene expression analysis of cDNA libraries using real-time qPCR-array

Individual cDNA libraries were generated from six IEC samples of patients with active CD, five IEC samples of patients with treated CD and five IEC samples of clinical controls (S1 Table) by reverse transcription of 400 ng total RNA (OD260/OD280 ratio ≥1.7) using random hexamers as templates for reverse transcription with the Superscript Vilo cDNA Synthesis Kit (Applied Biosystems, Foster City, CA). Real-time qPCR-array plates were custom designed with Taqman Gene Expression Assays for mRNAs of chemokines and their receptors (S2 Table). Each array included qPCR for 18S rRNA as housekeeping gene. Four ng of cDNA was used as template for each gene in the qPCR-array assays using 20 μL reaction mixture per gene according to the manufacturer’s instructions (TaqMan^™^ Array, Applied Biosystems). Emission from released reporter dye was monitored by the ABI prism 7700 sequence detection system (Applied Biosystems). mRNA concentrations were normalized to the18S rRNA concentration in the sample by calculating ΔCT between the CT for the mRNA species and the CT for 18S rRNA in the same sample (CT_mRNA of interest_—CT_18S rRNA_). Results are given as ΔCT or relative quantity (RQ) calculated as 2^(-ΔΔCT)^ where ΔΔCT is the ΔCT for the sample minus the median ΔCT-value for the IEC samples of clinical controls.

#### Gene expression analysis of total RNA using real-time qRT-PCR

Quantification of mRNAs was done in total RNA using the 3'-primer as template and recombinant thermostable *Thermus thermophilus* (Tth) DNA polymerase (Applied Biosystems) or Tth (LightCycler 480 RNA master hydrolysis probes, Cat. No. 04991885001; Roche, Mannheim, Germany) for gene specific reverse transcription in each qRT-PCR run and Taqman Gene Expression Assays with primers placed in different exons and a reporter dye marked probe placed over the exon boundary for the qPCR-step. This method has proven very sensitive and with minimal risk of signals from possible contamination by genomic DNA [6,28,29]. Emission from released reporter dye was monitored by the ABI prism 7700 sequence detection system. The mRNA concentrations were normalized to the18S rRNA concentration in the same sample by calculating the ΔCT between the CT for the mRNA species and the CT for 18S rRNA and results are given as RQ calculated as 2^(-ΔΔCT)^ where ΔΔCT is Δ CT for the sample minus the median of the ΔCT values of the IEC samples of control patients (n = 9), sham-treated enteroids (n = 4) and sham-treated tight monolayers (n = 3 or 4), respectively. All samples contained >25 U 18S rRNA per reaction mixture.

### Gene expression analysis at the protein level

Fresh duodenal biopsies were snap frozen in embedding medium (Tissue-tek O.C.T compound, Sakura Finetek, Zoeterwoude, Netherlands) and stored at -80°C until analysis of tissue distribution of gene products by indirect immunoperoxidase staining or two-color immunofluorescence staining.

#### Indirect immunoperoxidase staining

Cryostat sections (8 μm thick) were stained as earlier described [6,8]. The antibodies used were goat anti-human CX3CR1 polyclonal IgG (1:50, code K-13; Santa Cruz Biotechnology, Santa Cruz, CA), rabbit anti-human CXCL11 polyclonal IgG (1:50, code ab9955; Abcam, Cambridge, MA), rabbit anti-human SPINK4 polyclonal IgG mAb (1:50, clone EPR12492, IgG; Abcam), rabbit anti-human TFF1 mAb (1:50, clone EPR3972, IgG; Abcam), mouse anti-human MUC2 mAb (1:50, clone Ccp58, IgG1; Abcam), mouse anti-human ITLN1 mAb (1:25, clone 17, IgG1; Abcam) and mouse anti-human CX3CL1 mAb (1:10, clone EF12, IgG1; Santa Cruz Biotechnology). Concentration-matched irrelevant mouse mAb of IgG1 class (X0931, DakoCytomation, Glosterup, Denmark), goat IgG (code sc-2028; Santa Cruz Biotechnology) and rabbit IgG (code sc-2027; Santa Cruz Biotechnology) served as negative controls, and mouse anti-human CD45 mAb (clone 2B11, IgG1) and mouse anti-human epithelial cell antigen mAb (clone BerEP4, IgG1) as positive controls. Slides were inspected using an integrating, cooled color 3CCD camera (Color Chilled 3 CCD Hamamatsu Camera C5810; Hamamatsu Photonics, Hamamatsu City, Japan) on a standard light microscope attached to a computer. Images were captured with an interactive computer image analysis system (LeicaQWin; Leica Imaging Systems, Cambridge, UK). Immunohistochemistry with the different antibodies was performed in three independent experiments with 3 CD patients and 3 clinical controls. Tissue sections of a CD patient and a clinical control were placed next to each other on the slide, both sections enclosed by one paraffin-ring. Antibodies and other reagents were then added in one droplet covering both sections in order to obtain identical conditions. Capture of microscopic images was performed with the same setting throughout.

#### Two-color immunofluorescence staining

Cryostat sections (10 mm thick) of fresh frozen pieces of biopsies were air-dried, fixed in 4% paraformaldehyde for 15 min at room temperature and then rinsed in cold 0.02 mM PBS (pH 7.4). Sections were blocked and membranes permeabilized by incubation with PBS containing 0.1% Tween 20 and 10% normal horse serum (Sigma Aldrich) for 60 min. Thereafter the sections were incubated for 1 h with mouse anti-human epithelial cell antigen mAb (1:50; clone BerEP4, IgG1; DakoCytomation) together with rabbit anti-human BTN3A1 polyclonal IgG (1:5; Abcam) diluted in PBS containing 0.1% Tween 20 and 10% normal horse serum. The slides were washed and incubated for 60 min with goat anti-rabbit Alexa Fluore^®^ 488- conjugated secondary antibodies (1:100; IgG; Abcam). Thereafter incubated with PBS containing 0.1% Tween 20 and 10% normal horse serum. The slides were washed and incubated for 60 min with goat anti-mouse Alexa Fluore^®^ 594- conjugated secondary antibodies (1:100; IgG; Abcam). Slides were mounted using Slowfade Gold anti-fade reagent containing DAPI (Invitrogen). Microscopy was done using Nikon fluorescence microscope (Nikon Instruments Europe, Netherlands) and images were analyzed with NIS elements software. Three independent experiments were performed with 3 CD patients and 3 clinical controls. Staining of tissue sections of CD patients and controls, and capture of images were performed as described above.

### Statistical analysis

Statistical analyses of differences in mRNA expression levels for the different genes was done using two-sided Mann Whitney U-test when comparing IECs of CD patients and controls for signals in hybridization bead array, qPCR-array and qRT-PCR, one-way ANOVA with Dunett's compensation for multiple comparisons when comparing IFN-γ and IL-17A challenged enteroid cultures with sham-treated, and two-sided Student’s t test when comparing challenged tight monolayers and sham-treated. Statistical analyses were performed using the Prism 5 computer program (GraphPad Software, San Diego, CA). A *P*-value <0.05 was regarded as statistically significant.

### Ethics statement

Biopsies were taken as part of clinical examination. Written informed consent was obtained from the parents of participating children. The Regional Ethical Review Board in Umeå and the local Research Ethics Committee of the Faculty of Medicine, Umeå University approved the study, including the patient information and consent form (permit registration numbers: 96–304, 19970121; 04–159, 20090601; 2013/468-31).

## Results

In order to understand the role of IECs in the immunopathology of CD we focused on genes that showed increased expression in active disease. Initially we compared expression levels in purified IECs from duodenal mucosa of CD patients with active disease (IEC_CD_; n = 4) with purified IECs from control patients (IEC_CTR_; n = 4) using a genome-wide hybridization bead array for 18,401 genes. Contamination of IEC samples by IELs was estimated to 5–10%, as determined from the strength of the signal of six T lymphocyte specific genes (CD3D, CD3G, CD2, CD7, CD8A, CD247) in analysis of CD3^+^IELs of CD patients with active disease (n = 5; S1 File). Consequently 10% of the signal in CD3^+^IELs was subtracted from the signal of the corresponding gene in IEC samples before further analysis. The median expression mRNA levels in IEC_CD_, with and without correction for signals from contaminating CD3^+^IELs, and the relative quantity (RQ) calculated as the ratio between the median IEC_CD_-signal and the median IEC_CTR_-signal are shown in S1 File.

**Fig 1:**
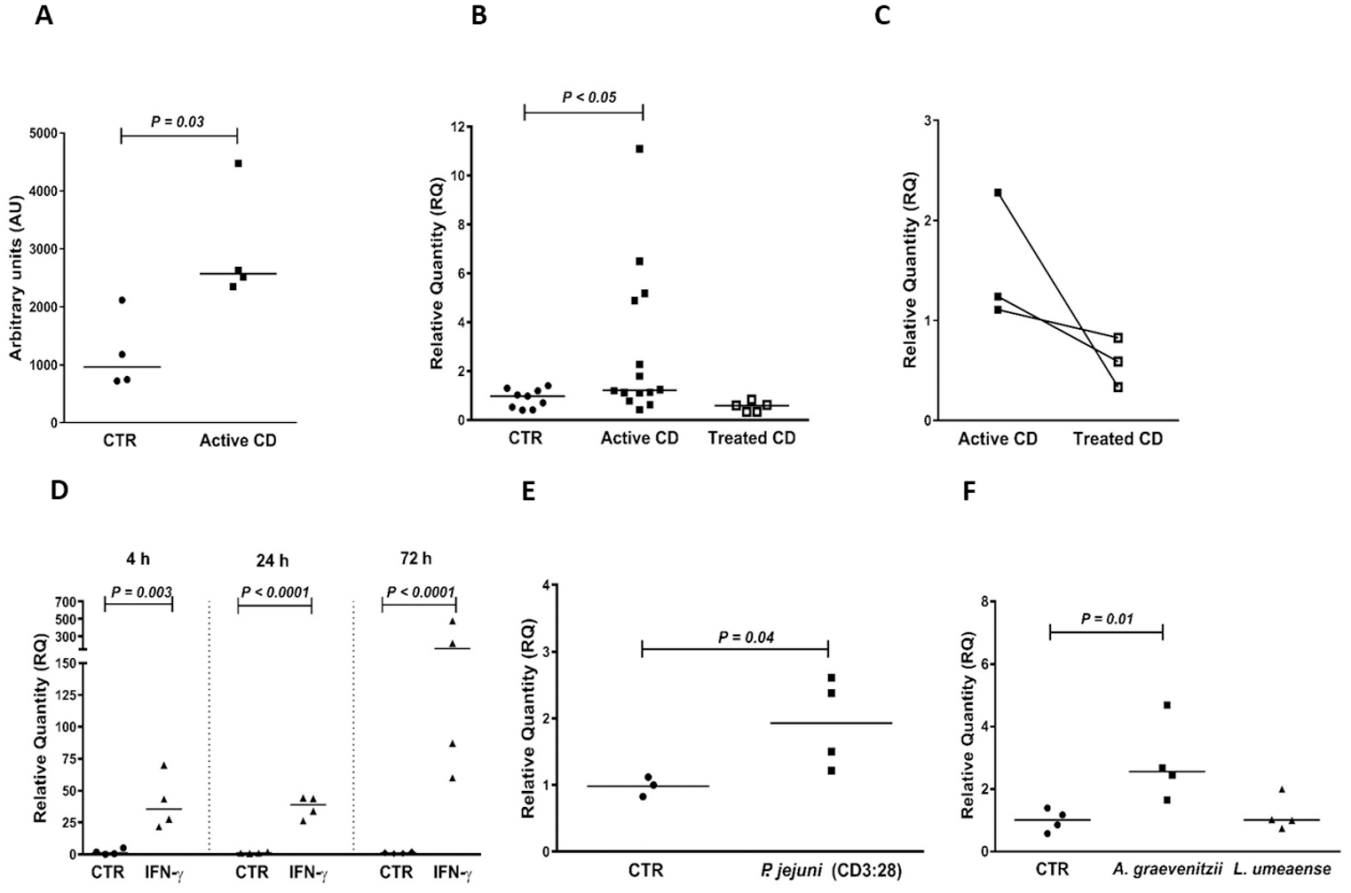
IRF1 is expressed at high levels in active CD and induced in IECs by both IFN-γ and CD associated bacteria. **(A)** IRF1 mRNA levels in IECs purified from duodenal biopsies of CD patients with active disease (Active CD) and controls (CTR) as estimated by genome-wide hybridization bead array. (**B**-**C**) IRF1 mRNA levels in IECs purified from duodenal biopsies of controls (CTR), patients with active CD (Active CD) and patients with inactive CD after >5 months on gluten-free diet (Treated CD) as estimated by qRT-PCR. **(D)** IRF1 mRNA levels as determined by qRT-PCR in T84 polarized tight monolayers incubated for 4 h, 24 h and 72 h with IFN-γ added to the basolateral side (IFN-γ) or sham-treated (CTR). (**E**-**F**) IRF1 mRNA levels in monolayers incubated for 4 h in anaerobic atmosphere with CD-associated bacteria added to the apical side in challenge medium or sham-treated incubated with challenge medium only (CTR). (**E**) *Prevotella jejuni* (*P*. *jejuni* (CD3:28)), (**F**) *Lachnoanaerobaculum umeaense* (*L*. *umeaense*) and *Actinomyces graevenitzii* (*A*. *graevenitzii*). (**A**-**C**) each point represents an individual patient. Lines in **(C)** connect the mRNA levels for the same patient biopsied before and after gluten-free diet. (**D**-**F**) each point represents an individual monolayer. In (**A**), mRNA levels are shown as signal strength in arbitrary units (AU). In (**B**-**F**), mRNA levels were normalized to the 18S rRNA content in the sample and calculated as relative quantity (RQ) by the 2^(-ΔΔct)^-method using the median Δct-value of controls and the median Δct-value of sham-treated monolayers as reference, for IECs and monolayers, respectively. *P*-values of statistically significant differences are given. Horizontal bars indicate medians.

**Fig 2:**
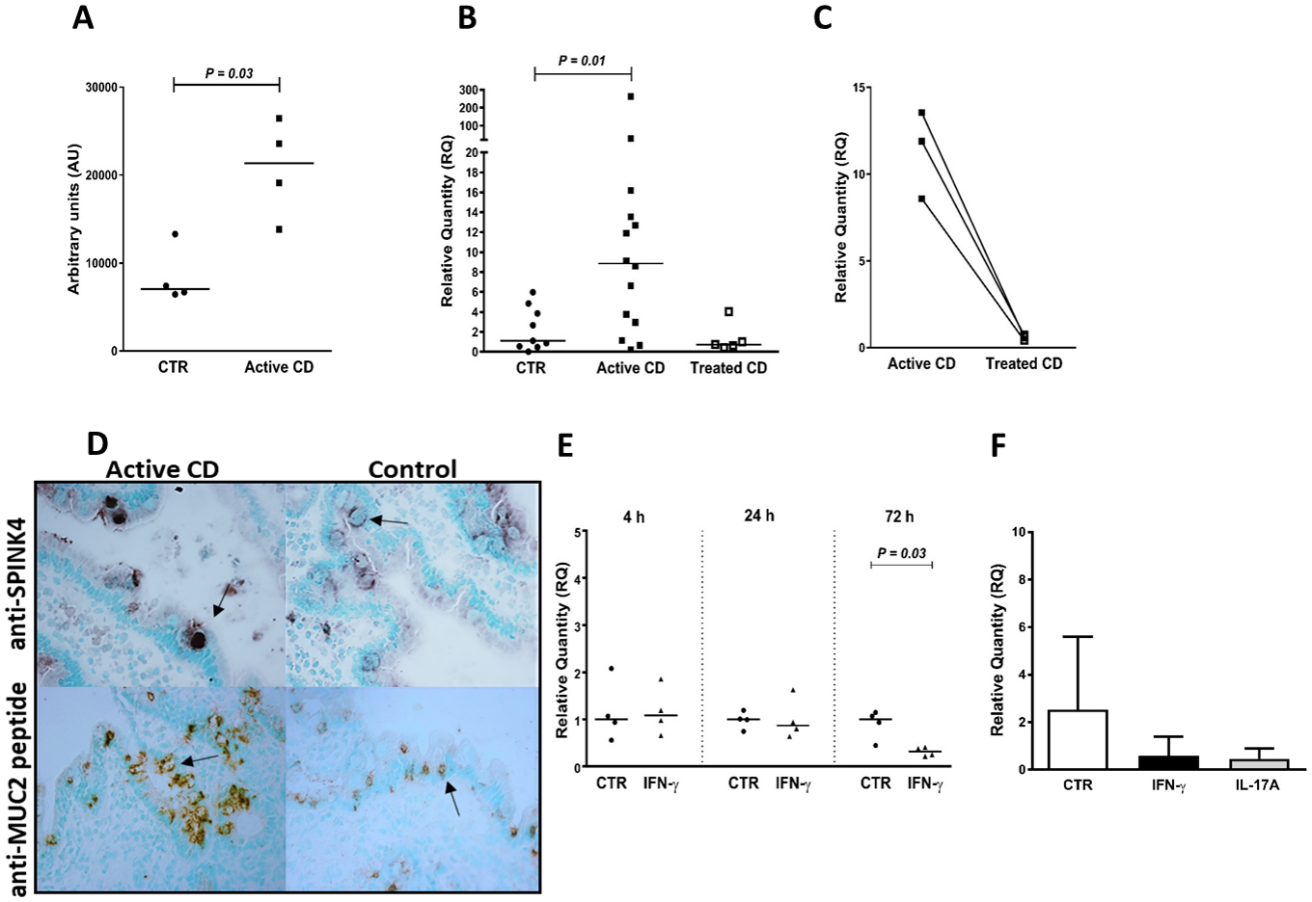
SPINK4 shows increased expression in goblet cells in active CD but is not induced by IFN-γ or IL-17A in IECs. **(A)** SPINK4 mRNA levels in IECs of CD patients with active disease (Active CD) and controls (CTR) as estimated by genome-wide hybridization bead array. **(B-C)** SPINK4 mRNA levels in IECs of controls (CTR), patients with active CD (Active CD) and patients with inactive CD (Treated CD) as estimated by qRT-PCR. **(D)** Immunohistochemical staining of duodenal mucosa of one of three CD patient with active disease (Active CD) and one of three controls (CTR) with anti-SPINK4 mAb and anti-MUC2-peptide mAb, respectively. The anti-SPINK4 mAb mainly stained the goblet of goblet cells. Note the more intense staining of goblets in the CD patient. Anti-MUC2 peptide mAb strongly stained the cytoplasm of mature goblet cells in both the CD patient and the control. In agreement with previously published results [18], the anti-MUC2 peptide mAb also stained enterocytes in the CD patient. Arrows indicate typical goblet cells. **(E)** SPINK4 mRNA levels in T84 polarized tight monolayers incubated for 4 h, 24 h and 72 h with IFN-γ (IFN-γ) or sham-treated (CTR). **(F)** SPINK4 mRNA levels estimated by qRT-PCR in short-term enteroid cultures established from duodenal biopsies of one clinical control and incubated for 24 h with IFN-γ or IL-17A added to the medium, i.e. at the basolateral side, or sham-treated (CTR) with 4 enteroid cultures per group. Columns indicate mean + 1 SD. For methods and statistics used see legend to Fig 1 and Material and methods.

**Fig 3:**
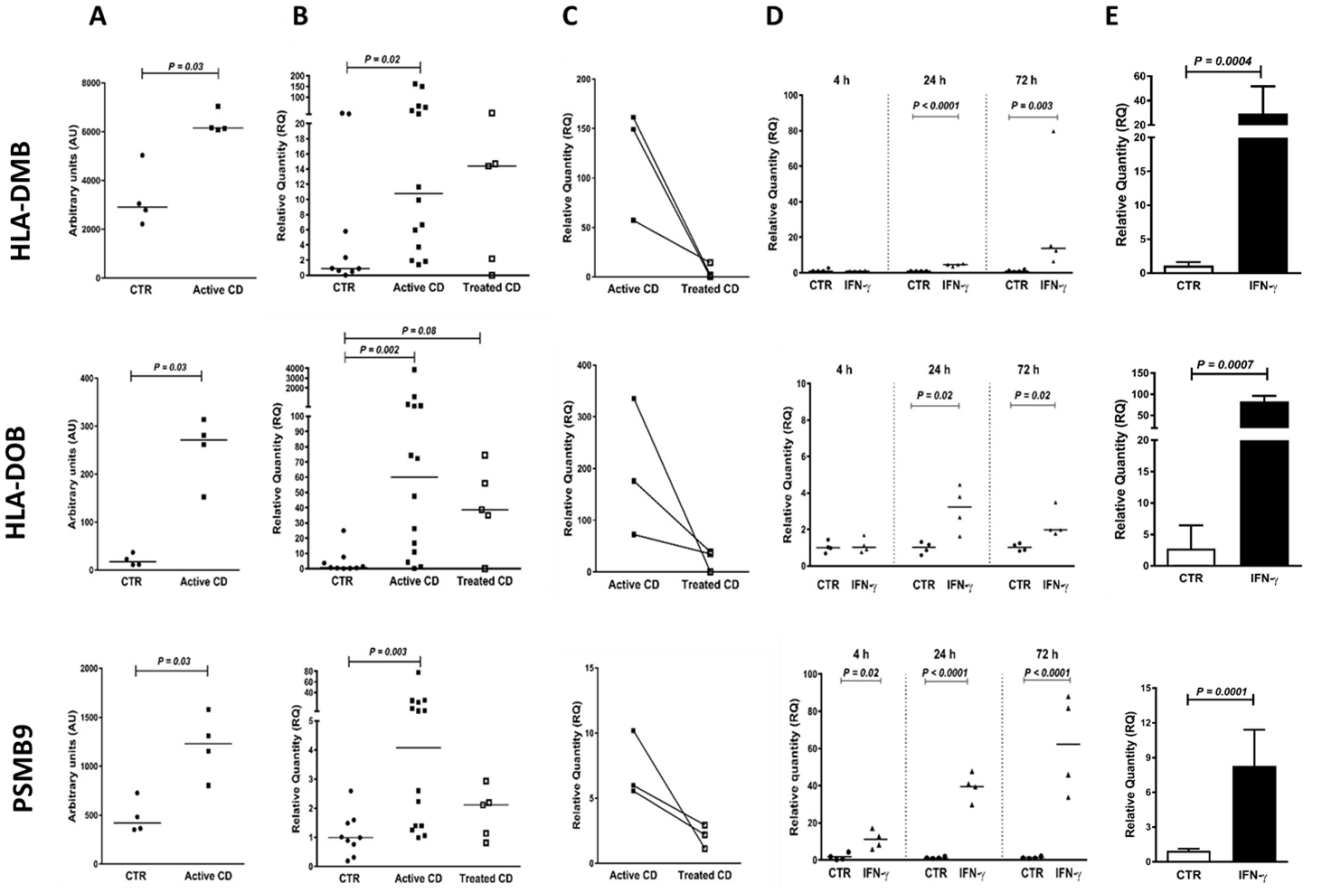
Genes involved in antigen presentation, i.e. HLA-DMB, HLA-DOB and PSMB9, are upregulated in active CD and slowly increased by IFN-γ stimulation. **(A)** Expression levels of HLA-DMB, HLA-DOB and PSMB9 mRNAs in IECs of CD patients with active disease (Active CD) and controls (CTR) as estimated by genome-wide hybridization bead array. **(B-C)** Expression levels of HLA-DMB, HLA-DOB and PSMB9 mRNAs in IECs of controls (CTR), patients with active CD (Active CD) and patients with inactive CD (Treated CD) as estimated by qRT-PCR. **(D)** Expression levels of HLA-DMB, HLA-DOB and PSMB9 mRNAs in T84 polarized tight monolayers treated for 4 h, 24 h and 72 h with IFN-γ (IFN-γ) or sham-treated (CTR). **(E)** Expression levels of HLA-DMB, HLA-DOB and PSMB9 mRNAs in short-term enteroid cultures incubated for 24 h with IFN-γ or IL-17A added to the medium or sham-treated (CTR). For methods and statistics used see legends to Figs 1 and 2 and Material and methods.

**Fig 4:**
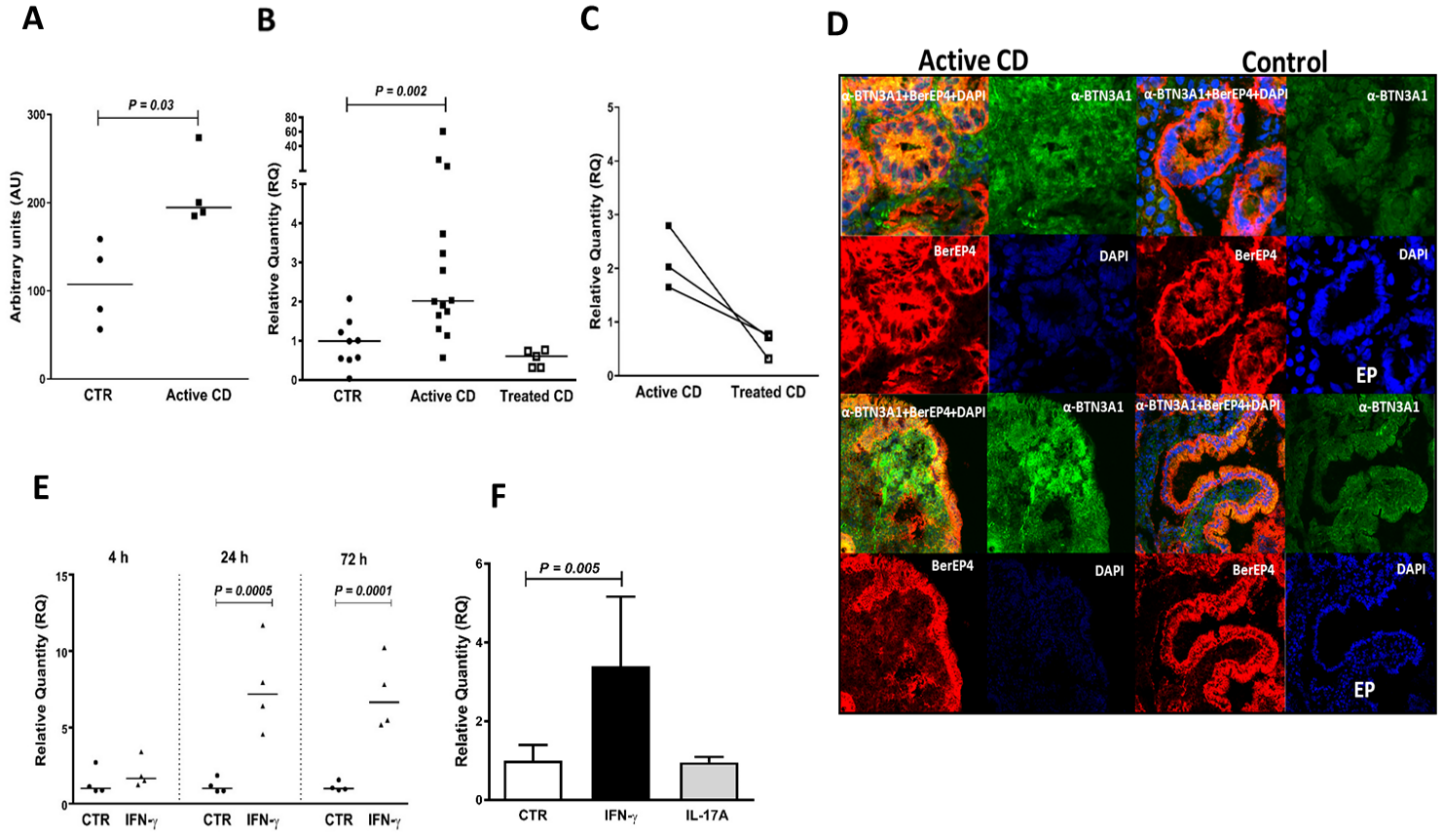
BTN3A1 is upregulated in IECs of patients with active CD and slowly induced in IECs by IFN-γ. **(A)** BTN3A1 mRNA levels in IECs of CD patients with active disease (Active CD) and controls (CTR) as estimated by genome-wide hybridization bead array. **(B-C)** BTN3A1 mRNA levels in IECs of controls (CTR), patients with active CD (Active CD) and patients with inactive CD (Treated CD) as estimated by qRT-PCR. **(D)** Immunofluoresce staining of duodenal mucosa of one of three CD patients with active disease (Active CD) and one of three controls (Control) for BTN3A1 by anti-BTN3A1 mAb (α-BTN3A1; green), for IECs by mAb BerEP4 (BerEP4; red), and for nuclei by 4´, 6-diamidino-2-phenylindole (DAPI; blue). The upper two rows show staining of the cryptal area and the lower two rows show staining of the luminal area in the CD patient and the villous area in the control. BTN3A1 and BerEP4 double-positive cells are yellow in overlays, i.e. micrographs marked α-BTN3A1+BerEP4+DAPI. Note the intense BTN3A1 staining of the cell membrane of IECs in the CD patient. **(E)** BTN3A1 mRNA levels in T84 polarized tight monolayers incubated for 4 h, 24 h and 72 h with IFN-γ (IFN-γ) or sham-treated (CTR). **(F)** BTN3A1 mRNA levels in short-term enteroid cultures incubated for 24 h with IFN-γ or IL-17A added to the medium or sham-treated (CTR). For methods and statistics used see legends to Figs 1 and 2 and Material and methods.

With a signal cut-off at 15 AU, the number of expressed genes in IEC_CTR_ was 10,297 and 478 of these genes were significantly higher in IEC_CD_ (*P* = 0.028 by two-sided, Mann-Whitney U-test). By analyzing RQ-values relative to frequencies of genes with a statistically significant difference between signals in IEC_CD_ and IEC_CTR_ it was found that only at RQ-values above 1.4 did the frequency of genes with a significantly higher signal in IEC_CD_ exceed 5%, i.e. the frequency expected at a *P*-vale of 0.05. Three hundred and sixty-three genes that showed a RQ >1.4 in the comparison IEC_CD_:IEC_CTR_ were statistically significant different. However, further data analysis and gene expression analysis by qRT-PCR of genes with low signals and RQs in the range 1.4 to 2.0 revealed that both signal strength and RQ was important to identify significant differences between the two groups and that genes with a signal <60 AU in IEC_CD_ and an RQ <1.8 could generally not be reproduced. Therefor we used a signal ≥60 AU and an RQ ≥1.8 as critieria for selection of genes of interest for further study. Totally 120 of the genes that fulfilled these criteria were significantly higher in IEC_CD_. They constituted 1.4% of all genes expressed at ≥60 AU and are listed in S3 Table. Genes are grouped into the categories 1) defense-related genes and 2) cellular housekeeping genes plus genes of unknown function(s), and arranged according to decreasing expression level within each category. Examples are shown in Figs 1A, 2A, 3A and 4A, and S1A, S2A and S3A Figs. Real-time qPCR-array analysis of 6 IEC_CD_ and 5 IEC_CTR_ samples for 39 chemokines and chemokine receptors (S2 Table) was performed as a supplementary screening to the hybridization bead array. These analyses revealed that CX3CL1 and CXCL11 were expressed at significantly higher levels in IEC_CD_ compared to IEC_CTR_ (S3 Table) in addition to the genes detected by hybridization bead array. IL-15 was expressed at very low levels in hybridization bead array (median and range 91 (70–110) and 82 (37–128) AU for IEC_CD_ and IEC_CTR_, respectively) and qPCR array (median ΔCTs were 23 and 22 for IEC_CD_ and IEC_CTR_, respectively). In agreement with previous studies [16,17] there was no increase in IL-15 mRNA levels in active disease (IEC_CD_:IEC_CTR_ ratio was 1.1 in hybridization bead array). Thus, other cells than IECs are the most likely the cellular source of IL-15 in active CD, as suggested by the results of León and coworkers who demonstrated IL-15 positive cells only in the lamina propria [30].

**Fig 5:**
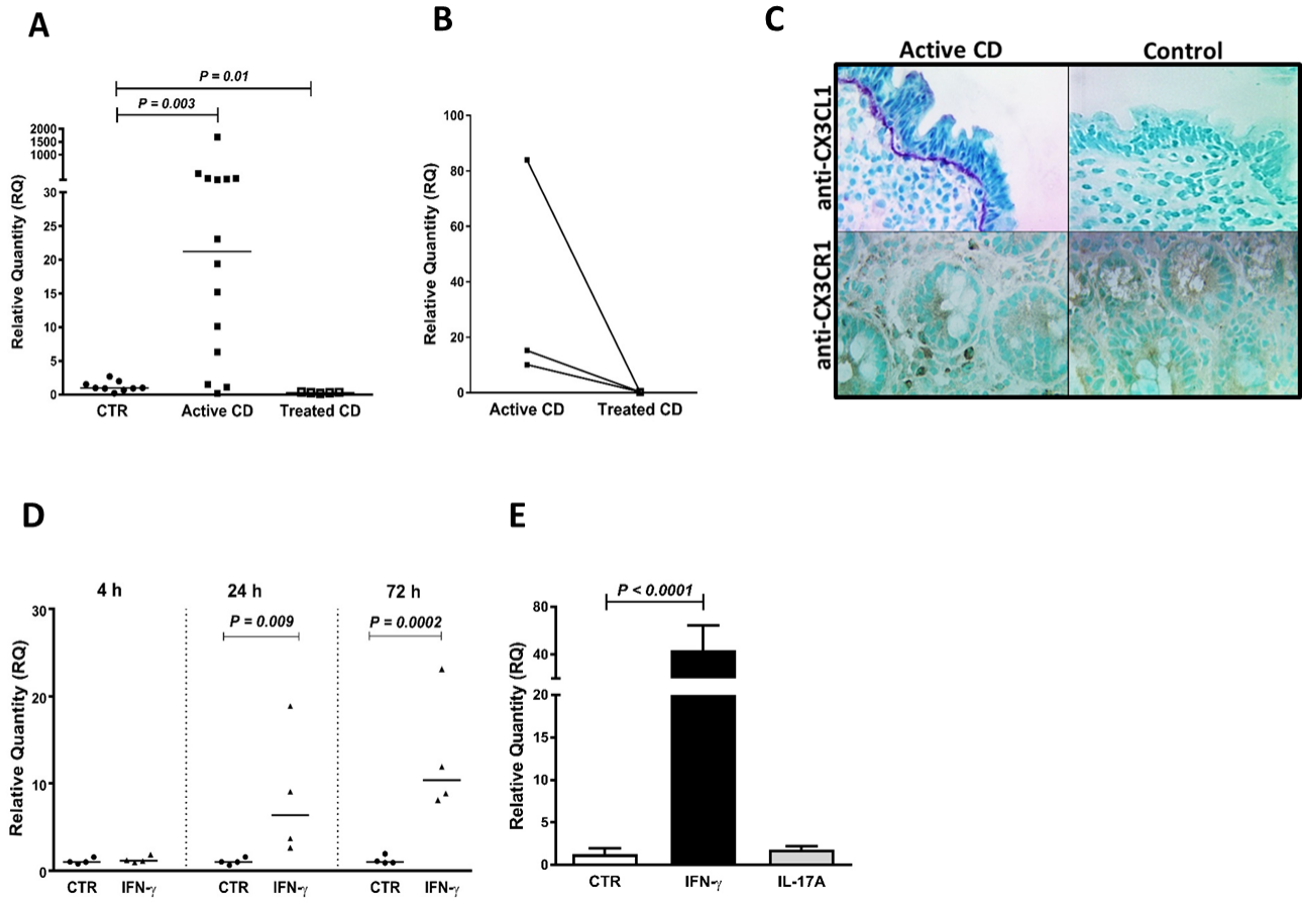
CX3CL1 is induced by IFN-γ in IECs, upregulated in IECs of patients with active CD and the secreted CX3CL1 accumulates at the basal membrane. **(A-B)** CX3CL1 mRNA levels in IECs of controls (CTR), patients with active CD (Active CD) and patients with inactive CD (Treated CD). **(C)** Immunohistochemical staining of duodenal mucosa of one of three CD patients with active disease (Active CD) and one of three controls (Control) with anti-CX3CL1 mAb (anti-CX3CL1) and polyclonal antibodies to its receptor CX3CR1 (anti-CX3CR1). Note staining for CX3CL1 of IECs and the basal lamina and CXC3R1^+^cells located juxtaposed to the epithelium in active CD. **(D)** CX3CL1 mRNA levels monolayers incubated for 4 h, 24 h and 72 h with IFN-γ (IFN-γ) or sham-treated (CTR). **(E)** CX3CL1 mRNA levels in short-term enteroid cultures incubated for 24 h with IFN-γ or IL-17A or sham-treated (CTR). For methods and statistics used see legends to Figs 1 and 2 and Material and methods.

In view of the uncertainty concerning the significance of higher signals in IEC_CD_ identified by the gene expression bead array we decided to use qRT-PCR with gene specific reverse transcription in Taqman Gene Expression assays to verify most defense-related genes in an enlarged set of purified IEC samples (14 patients with active CD, 5 patients with treated CD and 9 controls). Results are summarized in S3 Table and shown for the regulatory transcription factor interferon regulatory factor-1 (IRF1; Fig 1B), the serine peptidase inhibitor SPINK4 (Fig 2B), molecules involved in antigen loading on MHC class II (HLA-DMB and HLA-DOB) and MHC class I (PSMB9) (Fig 3B), the butyrophilin gene-family member BTN3A1 that regulates T lymphocyte proliferation and IFN-γ secretion, and is involved in antigen presentation to γδT cells (Fig 4B), the chemokine CX3CL1/fractalkine (Fig 5A), the galactofuranosyl binding intelectin-1 (ITLN1; S1B Fig), the mucous stabilizing trefoil factor-1 (TFF1; S2B Fig) and the class II MHC transactivator (CIITA; S3B Fig). A significantly higher mRNA level in IEC_CD_ compared to IEC_CTR_ (*P*<0.05) was confirmed in 25 of the 33 tested genes (76%) in the defense-related group, and 11 of the 14 tested genes (79%) in the other group. Thus, approximately 75% of the genes originally demonstrated to be significantly upregulated in IECs of CD patients with active disease could be confirmed using specific qRT-PCR assays and enlarged groups of CD patients and controls.

All genes that were upregulated in IECs of patients with active CD showed lower levels in IECs of CD patients who had been on gluten-free diet for more than 5 months (S3 Table). Notably, this was clearly demonstrated in the three individuals who donated biopsies both before and after treatment (Figs 1C, 2C, 3C, 4C and 5B and S1C, S2C and S3C Figs). With the exception of 8 genes, there was no significant difference between the expression levels in IECs of treated CD patients and controls. Levels of the virus defense molecule 2', 5'-oligoadenylate synthetase (OAS2) and the facilitative glucose transporter Solute Carrier Family 2 Member 10 (SLC2A10; also known as Glucose Transporter Type 10 (GLUT10)) were significantly higher in IECs of treated CD patients compared to clinical controls. HLA-DOB showed a similar trend (*P* = 0.08; Fig 3C). There was no correlation between anti-tTG2 serum concentrations and/or pathology score and gene expression levels (S4 Fig), suggesting that the higher level of these genes in CD patients is not caused by incomplete adherence to gluten-free diet. In contrast, levels of CX3CL1, CXCL10, ISG15, GBP2, SAM9DL, and SLC1A5 were lower, suggesting that gluten-containing diet causes an increase, although marginal, in IECs of controls.

Subsequently we addressed the question whether the cause(s) behind up-regulation of these genes is IFN-γ that is produced in large amounts by CD3^+^IELs in active CD (S1 File, and Forsberg et al. [6]) and/or IL-17A that is also produced by CD3^+^IELs in active CD, although less than IFN-γ [8]. Two *in vitro* models for human intestinal epithelium were used, T84 polarized tight monolayer cultures and short-term enteroid cultures developed from duodenal biopsies of children with normal upper endoscopic examination including histopathology (n = 6). In order to mimic the *in vivo* situation the cytokines were added to the basal side. Table 1 summarizes the results from IFN-γ and IL-17A stimulation on mRNA expression levels of 52 genes with confirmed up-regulation in active CD and Figs 1D, 2E, 2F, 3D, 3E, 4D, 4F, 5D and 5E, and S1E, S1F, S2E, S2F, S3D and S3E Figs show ten examples. Thirty-six of 52 genes (69%) were upregulated by IFN-γ, while only 4 of 45 genes (9%) were upregulated by IL-17A. With four exceptions, there was excellent agreement between results obtained in tight monolayers (adenocarcinoma of colon) and enteroids (normal duodenal mucosa). The exceptions were HLA-G, which was induced by IFN-γ in enteroids but not detected in monolayers, and NOS2A, IFI27, and GSDM that, in contrast, were upregulated by IFN-γ in monolayers but not in enteroids.

**Table 1:** Ability of IFN-γ and IL-17A to significantly upregulate mRNA levels of 38 genes that are expressed at increased levels in IECs of patients with active celiac disease as determined by qRT-PCR in the T84 polarized tight monolayer and the enteroid *in vitro* models. The protein expression of these genes in normal duodenal epithelium, as determined by immunohistochemistry, is also given.

Function	Gene ID	T84 tight monolayers^a^		Enteroids^a^		Expression in normal duodenum^b^
		IFN-γ	IL-17A	IFN-γ	IL-17A	
IFN-γ activation pathway in IECs	STAT1	Yes	No	Yes	No	Ent (+)
	IRF1	Yes		Yes	No	Ent (++)
T-cell stimulation and IFN-γ release	BTN3A1	Yes		Yes	No	Ent (++)
Adaptive immunity related	HLA-DMB	Yes		Yes		Ent (+)
	HLA-DOB	Yes		Yes(0)	No(0)	Goblet cells (+++)
	CIITA	Yes		Yes(0	No(0)	Ent (++)
	HLA-F	Yes				ND
	HLA-G	No(0)		Yes(0)	No; No(0) ^c^	No epithelial staining
	HLA-H/HFE	No		No	No	Ent (++)
	TAP1	Yes		Yes	No	Ent (+++), goblet cells (+++)
	UBA6	Yes				No epithelial staining
Proteosome/Immunoproteosome	PSMB9	Yes		Yes	No	Ent (+++)
	PSMC3	No	No	No	No	Ent (+++)
Chemokines and leukocyte migration	CXCL10	Yes				ND
	CXCL11	Yes				Ent (+++)
	CX3CL1	Yes	No	Yes	No; Yes	Ent (+++)
	CKLF	No	No	No	No	ND
	AIF1	No(0)		No(0)	No(0)	No epithelial staining
Inflammation	SAMD9	Yes				Ent (+++)
	SAM9DL	Yes				Ent (+++)
	PTGS2/COX2	No(0)		No; No(0)	No; No(0)	Ent (+)
Defense against bacteria and virus	ITLN1	Yes		No	No(d)	Goblet cells (+++)
	SPINK4	No(d)	No	No	No(d)	Goblet cell (+++), Ent (+)
	TFF1	No(d)	No	No	No	Goblet cell (+++)
	MUC2	No	No	No; No(0)	No; No(0)	Goblet cells (+++)
	NOS2A	Yes		No	No	Ent (+)
	DUOXA2	No(0)		No(0)	No(0)	Ent (++)
	GBP2	Yes		Yes	No	Ent (++)
	ISG15	Yes		Yes	No	Ent (+++)
	BST2	Yes	No			Ent (++)
	OAS2	Yes		Yes	No	Ent (+++)
	CASP1			Yes	No; Yes	Ent (+++)
	WAS	No	No	No	No	Ent (++)
	FER1L3	No	No	No	No	Ent (+++)
	IL18BP	Yes		Yes	No	Ent (+)
Immune function repressors	RNF31	Yes				Ent (+++)
	PRDM1	Yes				Ent (+)
Apoptosis	IFI27	Yes		No	No	Ent (++)
	XAF1	Yes		Yes	No	Ent (+++)
Angiogenesis promoting factor	ECGF1	Yes				No epithelial staining
Regulation of epithelial cell proliferation proliferation	GSDM	Yes		No	No	Ent (++)
G-protein coupled signal transduction	GNA14	No(d)	No	No; No(0)	No; No(0)	Caveolated cells (+++), Ent (+)
Translation	WARS	Yes				ND
Component of cellular motor protein	MYL4	Yes	No			No epithelial staining
Solute carrier	SLC1A5	No	No	No	No	Paneth cells (+++), Ent (+)
	SLC2A10	Yes				Ent (++)
	SLC12A8	No	No	No(d)	No	Ent (+++)
	SLC15A3	Yes	No			Ent (+++)
Unknown	EPSTI1	Yes				Ent (++)
	IFI35	Yes		Yes	No	ND
	C15ORF48	No	No	No; No(d)	Yes	Caveolated cells (+++), Ent (++)

^**a**^ Results from experiments in which 3 to 4 parallel cultures were stimulated with IFN-γ or IL-17A in parallel with 3 to 4 sham-treated control cultures. Each gene was analyzed in enteroid cultures from one to three donors. Statistical analysis was performed comparing the median Δct-value in the stimulated cultures to the median Δct-value in control cultures for the detection limit of the qRT-PCR assay for the respective gene. Results are given as:

Yes = Expression level statistically significantly upregulated in the indicated *in vitro* model (T84 tight monolayer or enteroid) by IFN-γ and IL-17A, respectively.

Yes(0) = Expression level statistically significantly induced from undetected in the indicated *in vitro* model (T84 tight monolayer or enteroid) by IFN-γ and IL-17A, respectively.

No = No statistically significant upregulation of expression level.

No(0) = Not detected either in stimulated or control cultures.

No(d) = Expression level statistically significantly decreased upon incubation with IFN-γ and IL-17A, respectively.

^b^ Protein expression of the indicated gene in human normal duodenal epithelium as evaluated from immunohistochemistry stained tissue sections published in the Human Protein Atlas (http://www.proteinatlas.org). Stained cell-types and staining intensity is indicated. Ent = enterocyte, columnar epithelial cell. Staining intensity: (+++) = very strong, (++) = strong, (+) = clear. ND = not done.

^c^ Different results in enteroids from different donors.

Fifteen genes that were upregulated in active CD, were not increased by neither IFN-γ nor IL-17A in any of the two *in vitro* models (Table 1). Is gluten, the CD-associated bacteria *Prevotella jejuni*, *Lachnoanaerobaculum umeaense* or *Actinomyces graevenitzii* [19–21] or a combination of these environmental factors responsible for up-regulation of any of these genes? We used tight monolayers for these analyses, since gluten and bacteria easily can be added to the apical side, i.e. the side at which environmental factors would primarily be expected to act *in vivo*. S4 Table summarizes the results. Most interestingly, IRF1 was upregulated by *P*. *jejuni* and *A*. *graevenitzii* (Fig 1E and 1F). None of the other 37 genes investigated was significantly upregulated by the bacteria and/or gluten.

Immunohistochemical data retrieved from Human Protein Atlas [31] of normal human duodenum (Table 1) revealed that almost all genes that were upregulated by IFN-γ are expressed in enterocytes. In contrast, several genes that were unaffected by IFN-γ and IL-17A are expressed in other epithelial cell-types. Thus, SPINK4, ITLN1, TFF1 and MUC2 were expressed in goblet cells, C15ORF48 and GNA14 in caveolated cells and SLC1A5 in Paneth cells. Previous immunohistochemical studies for MUC2 [18], revealed goblet cell metaplasia in active CD and was confirmed in the present study (Fig 2D). Thus, cells with the appearance of enterocytes were stained in addition to ordinary goblet cells. Paneth cell metaplasia was also demonstrated [18]. Two normally goblet cell-associated components, SPINK4 and ITLN1, were both expressed in enterocytes in the intestine of CD patients with active disease (Fig 2D and S1D Fig). It seems that upregulation of the seven genes mentioned above is intimately linked to the process of metaplasia.

We were not able to identify the inducing agent(s) for HLA-H/HFE, PSMC3, CKLF, PTGS2/COX2, DUOXA2, WAS, and SLC12A8, all of which are expressed in normal enterocytes (Table 1).

**Fig 6:**
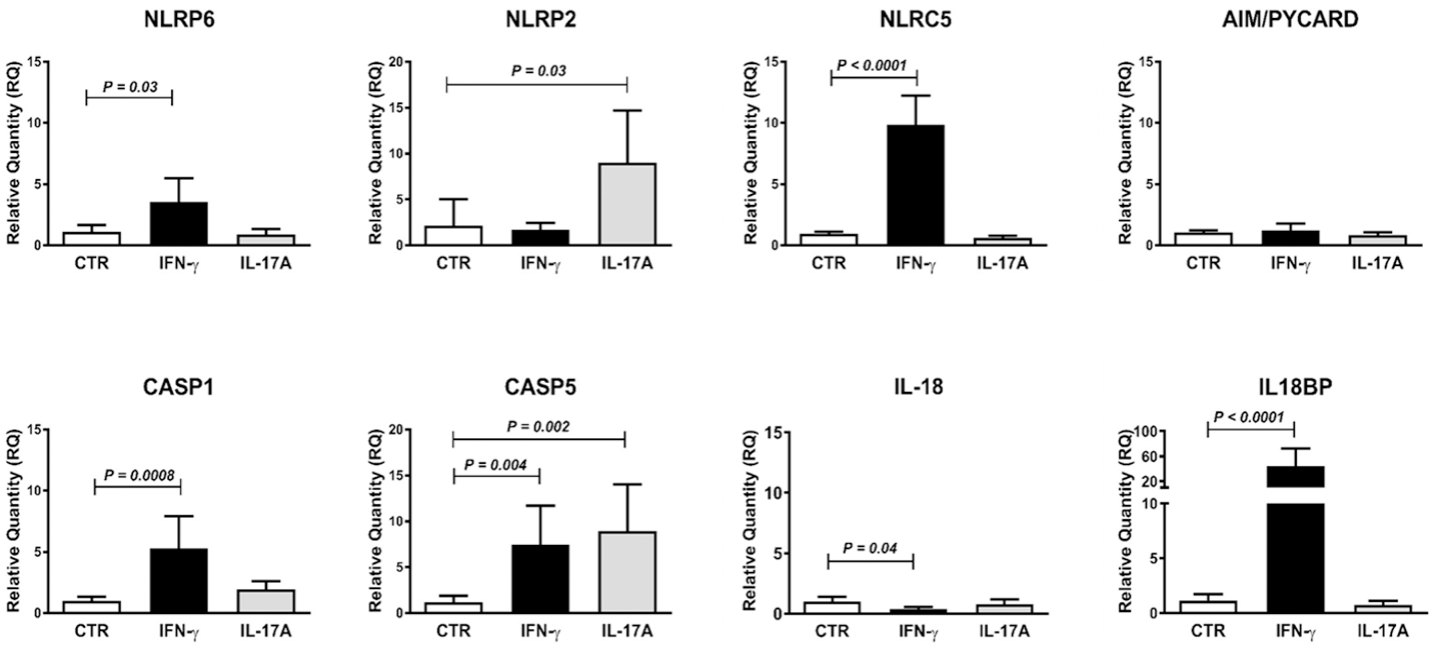
Expression levels of mRNAs for the inflammasome pattern recognition receptor molecules and caspase activators NLRP6 and NLRC5 as well as two the caspases, CASP1 and CASP5, are all increased by IFN-γ while IL-17A causes increase in expression levels of NLRP2 and CASP5 mRNAs. Expression levels of NLRP6, NLRP2, NLRC5, AIM/PYCARD, CASP1, CASP5, IL-18 and IL-18BP mRNAs in short-term enteroid cultures developed from duodenal biopsies of one clinical control and incubated for 24 h with IFN-γ or IL-17A added to the medium, i.e. at the basolateral side, or sham-treated (CTR) with 4 enteroid cultures per group. For methods and statistics used see legends to Figs 1 and 2 and Material and methods.

It is evident that IFN-γ plays a central role in the epithelial reaction in active CD and that CD3^+^IELs are the major producers [6]. What triggers the IFN-γ production? Signals from IECs are obvious candidates since IECs produce inflammasome cytokines in response to noxious agents and selected microorganisms. The inflammasome cytokine IL-18, also known as IFN-γ-inducing factor, is most likely the inducer. It is expressed at very high levels in IECs in active CD (median 10,760 AU) as determined by gene expression genome-wide hybridization bead array (S1 File) in contrast to inflammasome cytokine IL-1β that is expressed at low levels (median 167 AU). IL-18 mRNA was not upregulated in active CD. However, IL-18 is stored as an inactive protein, preIL-18, which is cleaved by caspase-1 (CASP1) into the biologically active form that can be secreted and bind to IL-18 receptors on IELs thereby inducing production of IFN-γ CASP1 mRNA is expressed at high levels in IECs (Table 2 and S3 Table) and increased in IFN-γ stimulated enteroids (Fig 6). mRNAs for the IL-18 receptor proteins are expressed in CD3^+^IELs and upregulated in active CD (median signal and RQ in CD3^+^IELs in active CD were 259 AU and 1.9 for IL18R1 mRNA and 709 AU and 4.0 for IL18RAP mRNA; S1 File). This suggests that active CD is characterized by production of high levels of biologically active IL-18, which in turn is responsible for production of IFN-γ by CD3^+^IELs. IECs can respond to IFN-γ. They express IFN-γ receptors (IFNRG1 mRNA at 2,333 AU; IFNRG2 mRNA at 3,006 AU), the signal-transducing complex (JAK1 mRNA at 250 AU; JAK2 mRNA at 233 AU), express and up-regulate STAT1 (mRNA at 4,106 AU) and the transcription factor IRF1 known to up-regulate pre-CASP1 (Fig 1A; S3 Table). Biologically active CASP1 is generated from pre-CASP1 through cleavage by AIM/PYCARD. mRNA for AIM is strongly expressed in IEC_CD_, although not at higher levels than in IEC_CTR_ (Table 2). To maintain high levels of AIM, inflammasome sensors need to be active. Consequently, we investigated which of the inflammasome sensors are expressed in IECs and enteroids of controls, and IECs of CD patients with active disease, and whether IFN-γ or IL-17A affects their expression. Only NLRP2, NLRP6, NLRP8 out of 10 known inflammasome forming sensors and 4 potentially inflammasome forming NLRs were expressed in IECs (Table 2). In enteroids, NLRP6 was expressed at high levels while NLRP2 and NLRP8 were barely detected (Table 2). NLRP6 was upregulated by IFN-γ and NLRP2 induced by IL-17A while neither IFN-γ nor IL-17A induced NLRP8 (Table 2 and Fig 6). NLRP6 is expressed in enterocytes and NLRP8 in goblet cells as assessed by immunohistochemistry [31]. NLPR2 was reported as a novel inflammasome sensor in astrocytes [32]. Hence, NLRP2 is likely to originate from neuroendocrine cells in IECs. The low levels of NLRP2 and NLRP8 in enteroides probably reflect low numbers of these cell-types, which is consistent with the low MUC2 levels (Table 2). It appears that NLRP6 and NLRP8 together form important inflammasome sensors in the defense of duodenal epithelium.

**Table 2:** Expression levels of inflammasome and related mRNAs in IECs of CD patients with active disease, IECs of controls, and enteroids established from normal human duodenum and effect of IFN-γ and IL-17A stimulation.

Gene ID	Expression level in			Statistically significant change in mRNA expression level in enteroids stimulated by	
	IEC_CD_^a^	IEC_CTR_^a^	Enteroids^b^	IFN-γ	IL-17A
*Inflammasome forming NLRs*					
NLRP1	<15	<15	0; 0	No	No
**NLRP2**	**334**	**110**	**1+; 0**	**No**	**Yes**
NLRP3	<15	<15	0; 0	No	No
**NLRP6**	**154**	**356**	**3+; 3+**	**Yes**	**No**
NLRP7	<15	<15			
**NLRP8**	**5,501**	**5,061**	**0; 0**	No	No
NLRP12	<15	<15			
NLRP13	<15	<15			
NLRC4/NAIP	<15	<15	0; 0	No	No
AIM2	<15	<15	0; 0		
*Non-inflammasome forming NLRs*					
NLRP10	25	31			
**NLRX1**	**177**	**181**	2+	No	No
**NLRC5 transactivator**	**364**	**70**	2+; 1+	Yes	No
*Unknown function(s) NLRs*					
NLRP4	<15	<15			
NLRP9	<15	<15			
NLRP11	<15	<15			
NLRP14	<15	<15			
*Non-NLR inflammasome related molecules*					
**AIM/PYCARD**	**2,998**	**2,517**	**3+; 2+**	**Yes; No**^d^	**No**
**CASP1**	**2,755**^c^	**1,546**	**3+; 2+**	**Yes**	**No**
CASP5	73	36	2+; 1+	Yes	Yes
**IL18**	**12,206**	**11,088**	**3+; 2+**	**No/down**	**No**
**IL18BP**	**157**	**83**	**1+; 1+**	**Yes**	**No**
IL1β	169	129	1+; 0	No	No
IL1RN	359	118	2+; 0	No	No
*Others*					
IL15	95	86			
TLR5	26	16	1+; 1+		
MUC2	4,848	2,333	0; 1+	No	No

^a^ Signal in gene expression hybridization bead array analysis expressed in arbitrary units. Uncorrected values.

^b^ Expression level as ΔCT-value (CT mRNA–CT 18S rRNA) determined by qRT-PCR. Categories: 3+ = ΔCT-values 3–5.9; 2+ = ΔCT-values 6–9.9; 1+ = ΔCT-values 10–19.9; 0 = ΔCT-values >20.0.

^c^ Statistically significantly higher in IEC_CD_ compared to IEC_CTR_.

^d^ Different result in enteroids from different donors.

## Discussion

The small intestinal mucosa is the primary target organ in CD. Here gluten peptides encounter the epithelium, penetrate through or between the enterocytes and induce a harmful adaptive immune response against gluten in individuals with the appropriate MHC class II haplotypes. Only a fraction of gluten-exposed individuals with these haplotypes will develop CD, strongly indicating that other factors are important for contracting and maintaining the disease. One major candidate is distorted functionality of the epithelial innate response, executed by IECs and IELs. A precipitating factor could be opportunistic microorganisms in the small intestinal microbiota. For example bacteria that at other sites of the body are kept at bay by competing microorganisms but in this less densely populated small intestinal environment can penetrate the protective mucous layer and interact with the IECs in such a way that partially digested gluten peptides can reach antigen presenting cells in the lamina propria and/or be inappropriately presented by IECs. That bacteria may play a role in CD is indicated by the following findings: 1) occurrence of CD in children has been shown to be epidemic [33,34] or have an endemic pattern [35]; 2) certain bacterial strains are associated to the small intestinal mucosa of children with CD [19–21]; 3) dysbiosis was demonstrated in the gut microbiota of CD patients [36,37].

**Fig 7:**
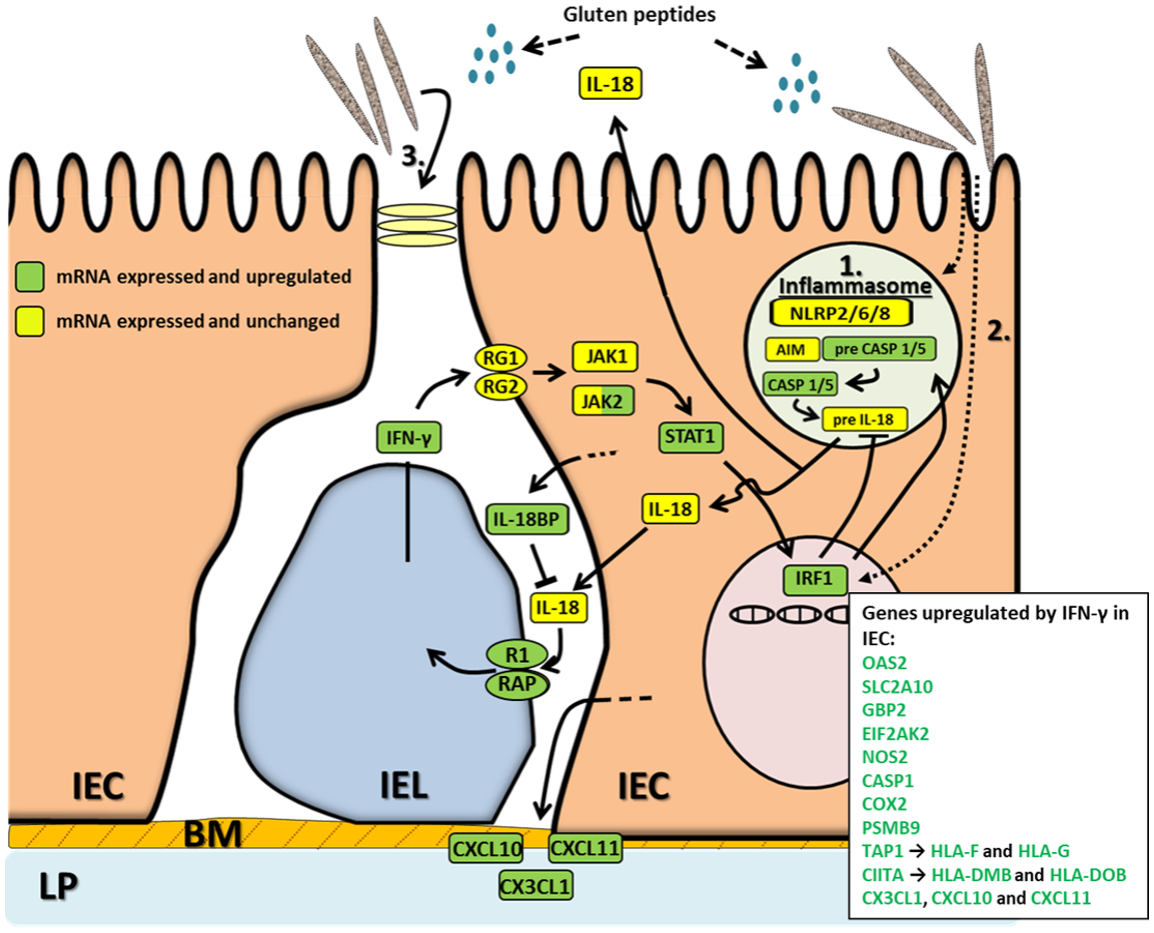
Hypothetical scenario of the initiating events of the inflammatory reaction at the small intestinal epithelium and the consequent crosstalk between IECs and IELs in active CD. Certain CD-associated bacteria, likely together with gluten peptides, can lead to IRF1 induction in IECs either (**1**) through the IL-18 dependent pathway of inflammasome activation in which stored pre-IL-18 is cleaved into biologically active IL-18 or (**2**) induce IRF1 directly which in turn up-regulates pre-CASP1, which is cleaved into its biologically active form with the aid of AIM and cleaves pre-IL-18. In both cases the mature IL-18 is secreted, binds to the IL-18 receptor (R1 and RAP) on IELs that consequently produce IFN-γ, which acts back on the IECs causing increased IRF1 levels and activation of a multitude of IFN-γ regulated genes, listed in the box in the left corner. **(3)** Constant high levels of IFN-γ results in inflammation and affects the tight junctions leading to a leaky epithelium with increased possibilities for paracellular passage of proteins, including gluten peptides, and even bacteria and their released products that can reach the lamina propria.

The results from the present study suggest that in CD, intestinal microbes, IECs and IELs interact to create a vicious IFN-γ-circle involving IRF1, the inflammasome sensors NLRP2/6/8, CASP1/5, IL-18, IL-18 receptor R1/RAP, IFN-γ receptor RG1/RG2, and STAT1 (Fig 7). Thus, we found that most defense-related genes upregulated in active CD are induced by IFN-γ (≈70%), the key cytokine in CD for which CD8^+^ αβIELs constitute the major cellular source [6,18]. Furthermore, the mRNA expression profile of IECs suggests that biologically active IL-18, which was previously shown to be present in the mucosa in active CD and mainly expressed in IECs [30,38], is the inducer of IFN-γ production by IELs through binding to the IL-18 receptor R1/RAP. Notably, the IL-18 receptor genes R1 and RAP were upregulated in CD3^+^IELs in active CD and IL-18 RAP shows genetic linkage to CD [39]. The IL-18 inhibitor IL18BP is also upregulated by IFN-γ and overexpressed in active CD suggesting that a normal feedback mechanism is over-ridden in CD, perhaps by continuous inflammasome activating signals from intestinal microbes via sensor molecules or directly via IRF1. Our data suggest a central role for IRF1 in generation of defense responses by IECs. That the high levels of IRF1 mRNA in active CD actually reflect increased IRF1 function is supported by the previous demonstration of increased IRF1 protein in IECs in active CD [40]. Analysis of possible sensor candidates for IL-18 inflammasome activation in IECs strongly pointed to NLRP6 and NLRP8 that both were expressed at high levels. NLRP2 and the flagellin binding Toll-like receptor 5 (TLR5) were expressed at low levels, while no other known sensors were detected.

Neither NLRP6 nor NLRP8 were expressed at different levels in IEC_CD_ compared to IEC_CTR_, indicating that the microbial sensor system in IECs of CD patients is normal and cannot explain the accumulation of *Prevotella* spp observed in the small intestine of CD patients [19]. In mice NLRP6 deletion resulted in overrepresentation of the *Prevotellaceae* family [41,42], which is inconsistent with our finding. CD associated bacteria must affect the IECs in another way. We found evidence for a direct effect on IRF1, leading to higher mRNA expression levels and presumably further up-regulation of the IFN-γ controlled genes (Fig 7). *P*. *jejuni* and *A*. *graevenitzii* but not *L*. *umeaense* caused up-regulation of IRF1, demonstrating species selectivity. How this is achieved is presently unknown and requires further studies. It is interesting to note that, in a recent publication, certain non-pathogenic reovirus were reported to activate IRF1 by a yet undefined mechanism leading to breakage of oral tolerance to dietary protein antigens [43]. Indications for an IFN-γ independent innate anti-bacterial response in active CD are the increased expression levels of the protease inhibitor SPINK4 and the antimicrobial lectin ITLN1. The induction was most pronounced in goblet cells suggesting that the mucous layer will be armed with these molecules. Increased expression of SPINK4 in CD has previously been reported [44].

IFN-γ activation of IECs lead to several changes that suggest gained features for immune defense via antigen presentation, both enhanced capabilities and altered repertoire on both MHC class I and MHC class II molecules and to both αβT cells and γδT cells. In active CD four IFN-γ induced, parallel changes in gene expression that could have implications for antigen presentation in the intestinal mucosa are observed:

Firstly, in active CD, there was elevated levels of CIITA, the master control factor for the expression of MHC class II genes, the beta-chain of HLA-DM (HLA-DMB), which governs peptide loading into the cleft of HLA-DR and HLA-DQ molecules [45], and the β-chain of HLA-DO (HLA-DOB), which modulates HLA-DM activity [45], while mRNAs for all chains of HLA-DR, HLA-DP and HLA-DQ were expressed at significant levels but not upregulated. IFN-γ stimulation caused an early response, within 4 hours, with induction of high levels of CIITA and a later response (≥24 hours) with increased levels of HLA-DMB and HLA-DOB. The results suggest that IECs normally gain increased capacity to present antigen to CD4^+^T-helper cells by IFN-γ stimulation and that the selection of presented T-cell epitopes is changed. It is tempting to speculate that low-grade antigen presentation under physiological conditions generates tolerance, while IFN-γ enhanced antigen presentation is part of immune responses in which IFN-γ has a role in initiating an adequate adaptive response. That IECs can present the protein antigen tetanus toxoid to specific CD4^+^T-helper cell clones was previously shown using T84 tight monolayers transfected with CIITA and HLA-DM [46]. Here we find that IFN-γ induces CIITA and HLA-DM expression in IECs suggesting that IFN-γ production in defense reactions by CD8^+^IELs enhances presentation of luminal protein antigens to CD4^+^IELs. HLA-DO inhibits HLA-DM mediated loading of peptides by competitive inhibition. The strong avidity of gliadin peptides to HLA-DQ2 and the fact that HLA-DO inhibition works better for HLA-DR than HLA-DQ, might lead to that gliadin peptides are presented also in the defense reaction consequently generating the adaptive immunity to them which is seen in CD patients.

Secondly, the proteasome component PSMB9 converts the proteasome complex to an immunoproteasome by causing changes in proteolytic activity that generate T-cell epitopes presented on MHC class I molecules to CD8^+^T-cells, a central mechanism in the defense to viral infection [47]. Both PSMB9 and transporter associated with antigen processing-1 (TAP1), which transport peptides across the endoplasmic reticulum into the membrane-bound compartment where loading of T-cell epitopes to MHC class I molecules takes place, showed elevated levels in active CD. Their levels were also strongly increased by IFN-γ stimulation in the two *in vitro* models, suggesting that the mechanisms of a typical anti-viral response with generation of cytotoxic T lymphocytes are activated in IECs of CD patients. Indeed, gliadin specific MHC class I restricted CD8^+^T cells have been demonstrated in the small intestinal mucosa of CD patients [13]. An additional sign of an anti-viral response-type in active CD was the strong elevation of OAS2, which functions as an inhibitor of viral replication.

Thirdly, levels of the MHC class I related molecule butyrophilin, BTN3A1, was elevated in active CD as well as in IFN-γ stimulated enteroids and tight monolayers. To the best of our knowledge this is the first time BTN3A1 is demonstrated in human small intestinal epithelial cells, shown to increase expression levels in such cells after IFN-γ stimulation, and last but not least shown to have significantly higher expression levels in IECs of CD patients with active disease compared to controls. Furthermore, the BTN3A1 protein was expressed in the cytoplasm and at the cell membrane of most IECs in active CD while it was barely detected in the epithelium of controls. BTN3A1 is known to present phosphoantigens to γδT cells, particularly those with the Vγ9 and Vδ2 T-cell receptor variable elements, a T-cell subtype that is present in the small intestinal epithelium constituting a few percent of the IELs [48,49]. Examples of natural phosphoantigens that are potent activators of these γδT cells are the endogenous isopentenyl pyrophosphate (IPP) that is overproduced in tumor cells and (*E*)-4-hydroxy-3-methyl-but-2-enyl pyrophosphate (HMBPP) that is secreted by many microbes. Thus, there is increased possibility of presentation of phosphoantigens of bacterial origin as well as stressed IECs in active CD leading to proliferation and secretion of IFN-γ [48]. Interestingly, it was recently reported that IL-18 strongly enhances the response of γδT cells to BNT3A1 dependent IPP and HMBPP stimulation [50], suggesting that IL-18 supported activation of intraepithelial γδT cells against bacterial, and possibly also endogenous, pyrophosphates on transiently expressed BTN3A1 is a factor that aggravates the mucosal inflammation in active CD.

Fourthly, enteroids responded to IFN-γ stimulation with strong induction of the chemokines CX3CL1, CXCL10 and CXCL11. All three were expressed at elevated levels in IECs in active CD. We previously demonstrated that the mRNA levels for these three chemokines are higher in small intestinal biopsies collected in active CD than in biopsies collected from the same patient with normalized mucosa after gluten-free diet [28]. Here we show that IECs constitute the major cellular source. Interestingly, we noted that the secreted CX3CL1 accumulates at the border between the epithelium and the lamina propria and that cells with macrophage morphology expressing the CX3CL1 receptor CX3CR1 are in close proximity to the epithelium in the mucosa of patients with active CD but not in controls. In mice, CX3CR1^+^ macrophages were shown to transfer protein antigen from the gut lumen to antigen presenting dendritic cells in the small intestinal lamina propria [51]. Thus, it is likely that there is increased sampling of peptides from the gut lumen in active CD executed by CX3CR1^+^ macrophages recruited to the epithelium by CX3CL1 thereby generating enhanced antigen presentation in the lamina propria. CXCL10 and CXCL11 recruit T lymphocytes and it has been shown that increased production of CXCL10 and CXCL11 leads to increased numbers of T lymphocytes in the intestinal mucosa of CD patients with active disease [52]. Consequently, in lamina propria the antigen load on dendritic cells is increased at the same time as the numbers of T lymphocytes increase. This milieu is rich in IFN-γ, which may shift the mode of action of the dendritic cells from tolerance inducing to T-helper cell inducing thereby creating possibilities for induction of adaptive immunity to luminal protein antigens locally. By this way gliadin specific CD4^+^T-helper cells may be generated leading to accumulation of memory T cells in the lamina propria that gives CD the chronic feature by specifically responding to each boost by dietary gluten with a misdirected Th1 response that causes inflammation and maintains the disease.

Three of the IFN-γ induced genes, namely OAS2, SLC2A10 and HLA-DOB remained expressed at higher levels in IECs of patients with treated CD than in IECs of controls. The treated CD group included only 5 patients. Thus, further studies are required to confirm the findings. There are at least three, not mutually exclusive, tentative explanations for why the expression levels are higher in CD patients than controls. 1) High expression levels of these three genes could be an inherent risk factor predisposing for contraction of the disease. Since HLA-DO, via HLA-DM, directs selection of peptides presented in MHC class II molecules at the cell surface, IECs with an elevated HLA-DOB level might display different antigens than normally and pave the way for undesired immune responses, e.g. against gluten peptides. 2) IFN-γ stimulation caused increased expression levels of all three genes in the *in vitro* models. Previously we showed that IFN-γ levels of CD3^+^IELs do not fully normalize in treated CD patients [6]. It is possible that OAS2, SLC2A10 and/or HLA-DOB are responsive to IFN-γ at low concentrations and that the IFN-γ concentrations in the epithelium of treated CD patients are enough to trigger increased expression. 3) There could be something in the gluten-free diet that activates these genes in CD patents. In a double-blind intervention study we showed that a substantial fraction of pediatric CD patients do not tolerate oats, estimated as normalization of expression levels of genes involved in the immunity in small intestinal mucosa [28]. Unfortunately were OAS2, SLC10A2 and HLA-DOB not analyzed and therefor it is not know whether oats in the diet influence expression levels of these genes. Further, we do not know if the patients in the treated CD group had oats in their gluten-free diet, however, in Sweden this is common. Nonetheless, it is an interesting possibility that the treated CD patients in the present study belong to the fraction of patients that do not tolerate oats, had oats in their gluten-free diet and therefor had high levels of these genes caused by intake of oats. Furthermore, high levels of OAS2 might reflect a viral component of CD. OAS2 exerts it anti-viral effect by causing degradation of viral and cellular RNA thereby decreasing the protein synthesis in the cell. Longstanding dampened protein synthesis might hamper cell function in both infected and non-infected cells in CD patients.

It is clear from these studies that the epithelium is far from dead or dying in CD, rather it is very active. The gene expression profile in IEC_CD_ compared to IEC_CTR_ and the IFN-γ responses of enteroids of normal IECs indicate that active CD is an exorbitant version of the immune response normally executed at the epithelial lining. The key factor appears to be over-expression of IRF1 that could be inherent and/or due to presence of undesirable microbes like *P*. *jejuni* or certain reovirus that act directly on IRF1. Dual activation of IRF1 and IRF1-regulated genes, both directly and via the IL-18 dependent inflammasome would drastically enhance the inflammatory response and lead to the pathological situation seen in active CD.

## Supporting information

S1 Fig Intelectin-1, ITLN1, shows increased expression in IECs in active CD but is not induced by IFN-γ or IL-17A in IECs. **(A)** Expression levels of ITLN1 mRNA in IECs purified from duodenal biopsies of CD patients with active disease (Active CD) and clinical controls (CTR) as estimated by genome-wide hybridization bead array. Each point represents an individual patient. **(B-C)** Expression levels of ITLN1 mRNA in IECs isolated from fresh biopsies of small intestinal mucosa of clinical controls (CTR), patients with active CD (Active CD) and patients with inactive CD after more than 5 month on a gluten-free diet (Treated CD). Each point represents an individual patient. Lines in **(C)** connect the mRNA levels for the same CD patient biopsied before and after gluten-free diet. **(D)** Immunohistochemical staining of duodenal mucosa of one CD patients with active disease (Active CD) and one clinical controls (CTR) with anti-ITLN1 mAb. Staining for ITLN1 is seen at the apical side of IECs in both controls and CD patients but with much higher intensity in active CD. Micrographs show representative results of three independent experiments with 3 patients per group. **(E)** Expression levels of ITLN1 mRNA in T84 polarized tight monolayers treated for 4 h, 24 h and 72 h with IFN-γ added to the basolateral side (IFN-γ) or sham-treated (CTR). Each point represents one monolayer. **(F)** Expression levels of ITLN1 mRNA in short-term enteroid cultures established from duodenal biopsies of one clinical control and incubated for 24 h with IFN-γ or IL-17A added to the medium, i.e. at the basolateral side, or sham-treated (CTR) with 4 enteroid cultures per group. Horizontal bars indicate medians. Columns indicate mean + 1 SD. For methods and statistics used see legends to Figs 1 and 2 and Material and methods.
(TIF)

S2 Fig Trefoil factor-1, TFF1, shows increased expression in goblet cells in active CD but is not induced by either IFN-γ or IL-17A in IECs. **(A)** Expression levels of TFF1 mRNA in IECs of CD patients with active disease (Active CD) and clinical controls (CTR) as estimated by genome-wide hybridization bead array. **(B-C)** Expression levels of TFF1 mRNA in IECs of clinical controls (CTR), patients with active CD (Active CD) and patients with inactive CD (Treated CD). **(D)** Immunohistochemical staining of duodenal mucosa of one of three CD patients with active disease (Active CD) and one of three clinical controls (CTR) with anti-TFF1 mAb. Staining for TFF1 is seen in goblet cells in CD patients with active disease but not in controls. **(E)** Expression levels of TFF1 mRNA in T84 polarized tight monolayers treated for 4 h, 24 h and 72 h with IFN-γ (IFN-γ) or sham-treated (CTR). **(E)** Expression levels of TFF1 mRNA in short-term enteroid cultures established from one clinical control and incubated for 24 h with IFN-γ or IL-17A added to the medium or sham-treated (CTR) with 4 enteroid cultures per group. For methods and statistics used see legends to Figs 1 and 2 and Material and methods.
(TIF)

S3 Fig CIITA, the class II, major histocompatibility complex, transactivator is induced by IFN-γ and significantly upregulated in active CD. **(A)** Expression levels of CIITA mRNA in IECs of CD patients with active disease (Active CD) and clinical controls (CTR) as estimated by genome-wide hybridization bead array. **(B-C)** Expression levels of CIITA mRNA in IECs isolated from fresh biopsies of small intestinal mucosa of clinical controls (CTR), patients with active celiac disease (Active CD) and patients with inactive celiac disease (Treated CD) as estimated by qRT-PCR. **(D)** Expression levels of CIITA mRNA in T84 polarized tight monolayers treated for 4 h, 24 h and 72 h with IFN-γ (IFN-γ) or sham-treated (CTR). **(F)** Expression levels of CIITA mRNA in short-term enteroid cultures established from one clinical control and incubated for 24 h with IFN-γ or IL-17A added to the medium or sham-treated (CTR). For methods and statistics used see legends to Figs 1 and 2 and Material and methods.
(TIF)

S4 Fig Expression levels of OAS2, SLC2A10 and HLA-DOB mRNAs in IECs of treated CD patients on gluten-free diet do not correlate with serum concentrations of anti-tTG2 IgA or histopathological score. Expression levels, as estimated by qRT-PCR, of OAS2, SLC2A10 and HLA-DOB in IECs of CD patients on a gluten-free diet. Each dot represents IECs of one individual. Black dots represents IECs of patients with serum concentration of anti-tTG2 IgA ≤ 6 U/mL and pathological score Marsh 0 and red dots represents IECs of patients with anti-tTG2 IgA concentrations > 6 U/mL and pathological score Marsh 0/1 or 1. For clinical details see S1 Table. For methods used see legend to Fig 1 and Material and methods.
(TIF)

S1 Table Description of study subjects. (DOCX)

S2 Table List of TaqMan gene expression assays utilized in qRT-PCR and qPCR array. (XLSX)

S3 Table Genes with significantly higher mRNA levels in IECs of CD patients with active disease compared to IECs from control patients determined by genome-wide hybridization bead array for gene expression and confirmed by qRT-PCR. (XLSX)

S4 Table Ability of gluten and CD-associated bacteria to significantly up-regulate mRNA levels of 38 genes expressed at increased levels in IECs of CD patients with active disease as determined in the T84 tight monolayer *in vitro* model by gene expression hybridization bead array and qRT-PCR analysis. (XLSX)

S1 File Gene expression assessed by genome wide hybridization bead array in IECs and CD3^+^IELs isolated from small intestinal biopsies of CD patients with active disease. (XLSX)

S2 File ΔCT-values and RQ-values of qRT-PCR analyses for gene expression level determination in of IEC samples, polarized tight monolayers and enteroids. (XLSX)
