# Supplementary figures and images for: Immunopathology of childhood celiac disease—Key role of intestinal epithelial cells
Source: PLoS One. 2017 Sep 21;12(9):e0185025. doi: 10.1371/journal.pone.0185025 (PMC5608296; doi:10.1371/journal.pone.0185025)

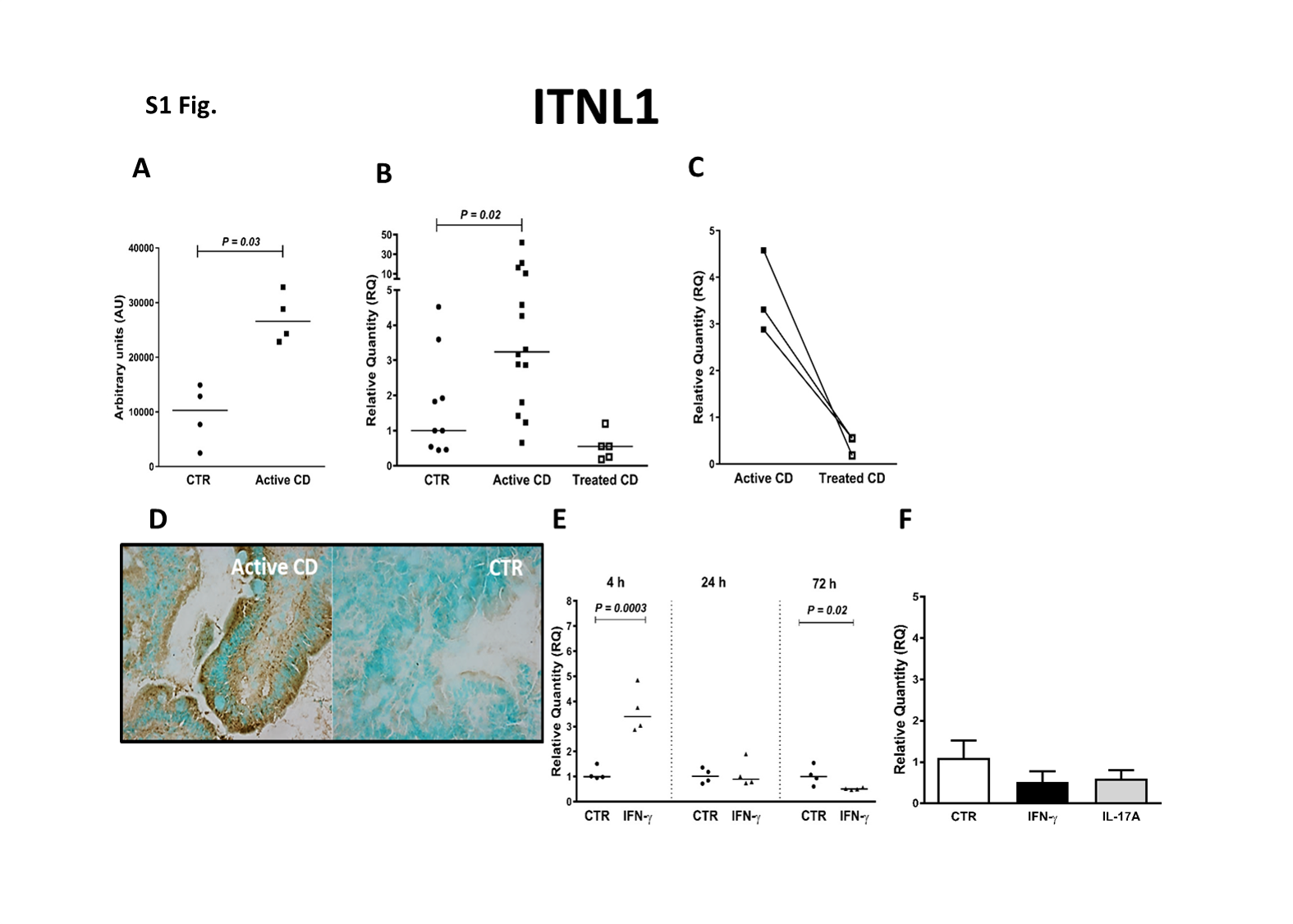

Supplement: S1 Fig — (A) Expression levels of ITLN1 mRNA in IECs purified from duodenal biopsies of CD patients with active disease (Active CD) and clinical controls (CTR) as estimated by genome-wide hybridization bead array. Each point represents an individual patient. (B-C) Expression levels of ITLN1 mRNA in IECs isolated from fresh biopsies of small intestinal mucosa of clinical controls (CTR), patients with active CD (Active CD) and patients with inactive CD after more than 5 month on a gluten-free diet (Treated CD). Each point represents an individual patient. Lines in (C) connect the mRNA levels for the same CD patient biopsied before and after gluten-free diet. (D) Immunohistochemical staining of duodenal mucosa of one CD patients with active disease (Active CD) and one clinical controls (CTR) with anti-ITLN1 mAb. Staining for ITLN1 is seen at the apical side of IECs in both controls and CD patients but with much higher intensity in active CD. Micrographs show representative results of three independent experiments with 3 patients per group. (E) Expression levels of ITLN1 mRNA in T84 polarized tight monolayers treated for 4 h, 24 h and 72 h with IFN-γ added to the basolateral side (IFN-γ) or sham-treated (CTR). Each point represents one monolayer. (F) Expression levels of ITLN1 mRNA in short-term enteroid cultures established from duodenal biopsies of one clinical control and incubated for 24 h with IFN-γ or IL-17A added to the medium, i.e. at the basolateral side, or sham-treated (CTR) with 4 enteroid cultures per group. Horizontal bars indicate medians. Columns indicate mean + 1 SD. For methods and statistics used see legends to Figs 1 and 2 and Material and methods. (TIF) [file pone.0185025.s001.tif]

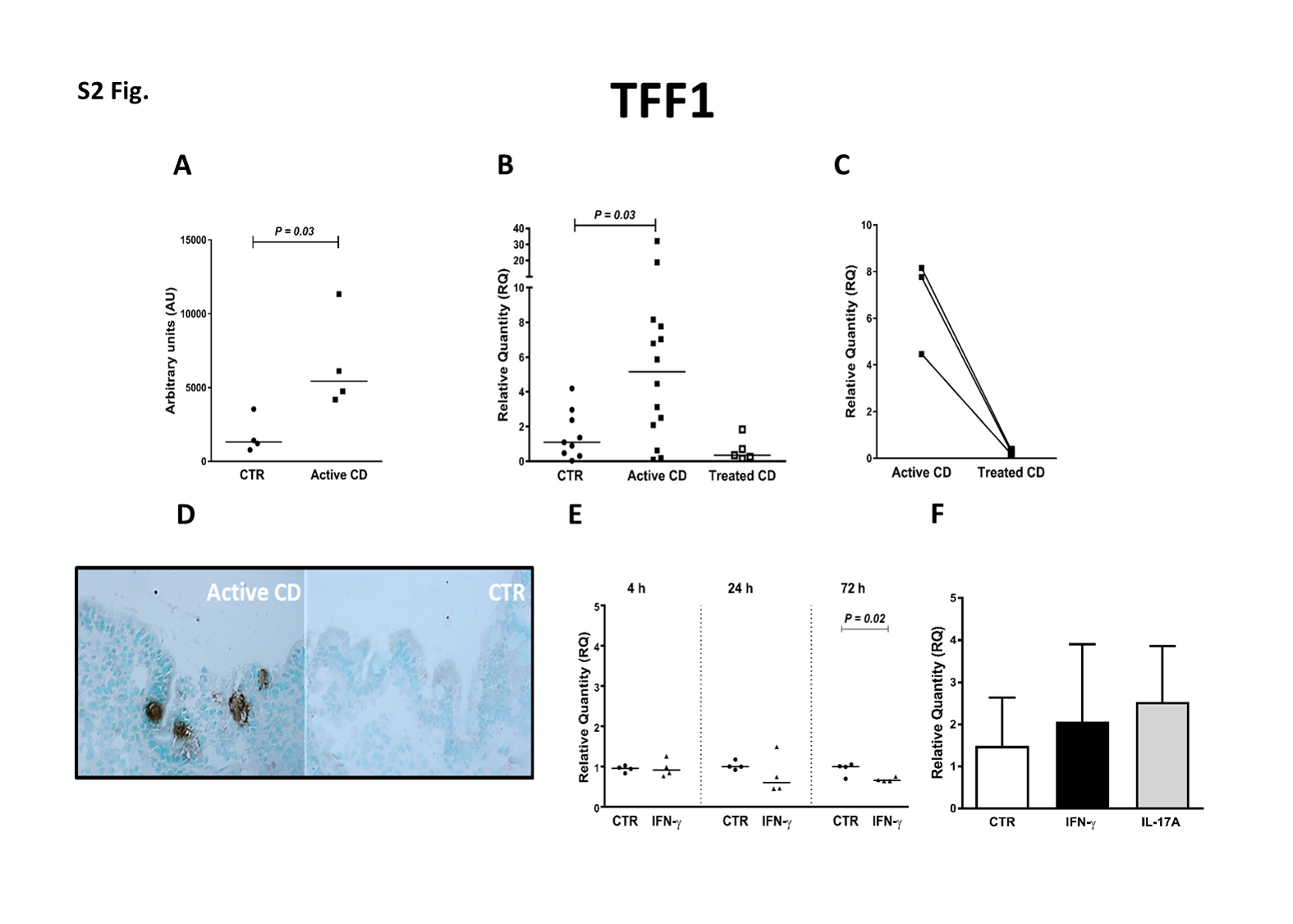

Supplement: S2 Fig — (A) Expression levels of TFF1 mRNA in IECs of CD patients with active disease (Active CD) and clinical controls (CTR) as estimated by genome-wide hybridization bead array. (B-C) Expression levels of TFF1 mRNA in IECs of clinical controls (CTR), patients with active CD (Active CD) and patients with inactive CD (Treated CD). (D) Immunohistochemical staining of duodenal mucosa of one of three CD patients with active disease (Active CD) and one of three clinical controls (CTR) with anti-TFF1 mAb. Staining for TFF1 is seen in goblet cells in CD patients with active disease but not in controls. (E) Expression levels of TFF1 mRNA in T84 polarized tight monolayers treated for 4 h, 24 h and 72 h with IFN-γ (IFN-γ) or sham-treated (CTR). (E) Expression levels of TFF1 mRNA in short-term enteroid cultures established from one clinical control and incubated for 24 h with IFN-γ or IL-17A added to the medium or sham-treated (CTR) with 4 enteroid cultures per group. For methods and statistics used see legends to Figs 1 and 2 and Material and methods. (TIF) [file pone.0185025.s002.tif]

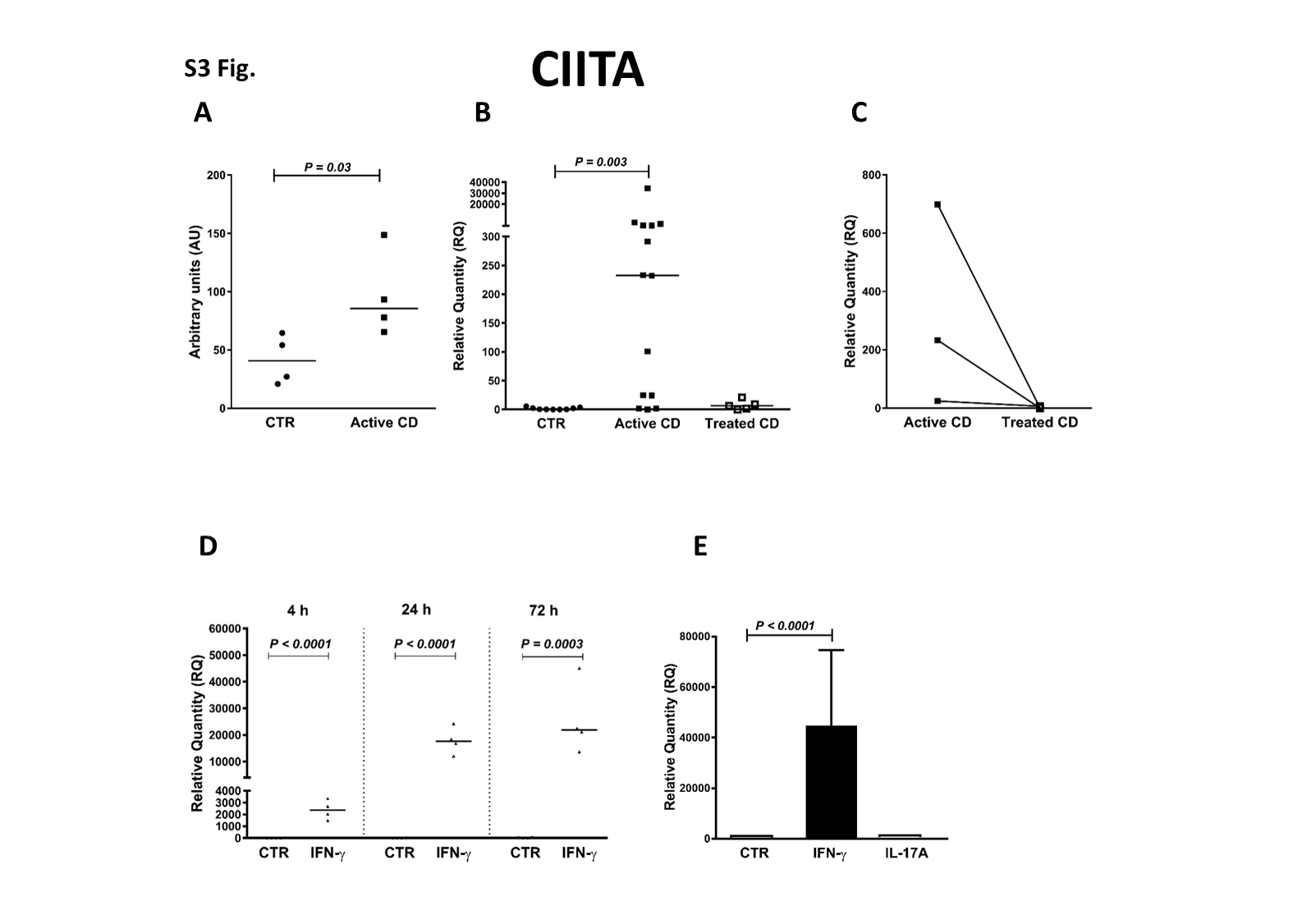

Supplement: S3 Fig — (A) Expression levels of CIITA mRNA in IECs of CD patients with active disease (Active CD) and clinical controls (CTR) as estimated by genome-wide hybridization bead array. (B-C) Expression levels of CIITA mRNA in IECs isolated from fresh biopsies of small intestinal mucosa of clinical controls (CTR), patients with active celiac disease (Active CD) and patients with inactive celiac disease (Treated CD) as estimated by qRT-PCR. (D) Expression levels of CIITA mRNA in T84 polarized tight monolayers treated for 4 h, 24 h and 72 h with IFN-γ (IFN-γ) or sham-treated (CTR). (F) Expression levels of CIITA mRNA in short-term enteroid cultures established from one clinical control and incubated for 24 h with IFN-γ or IL-17A added to the medium or sham-treated (CTR). For methods and statistics used see legends to Figs 1 and 2 and Material and methods. (TIF) [file pone.0185025.s003.tif]

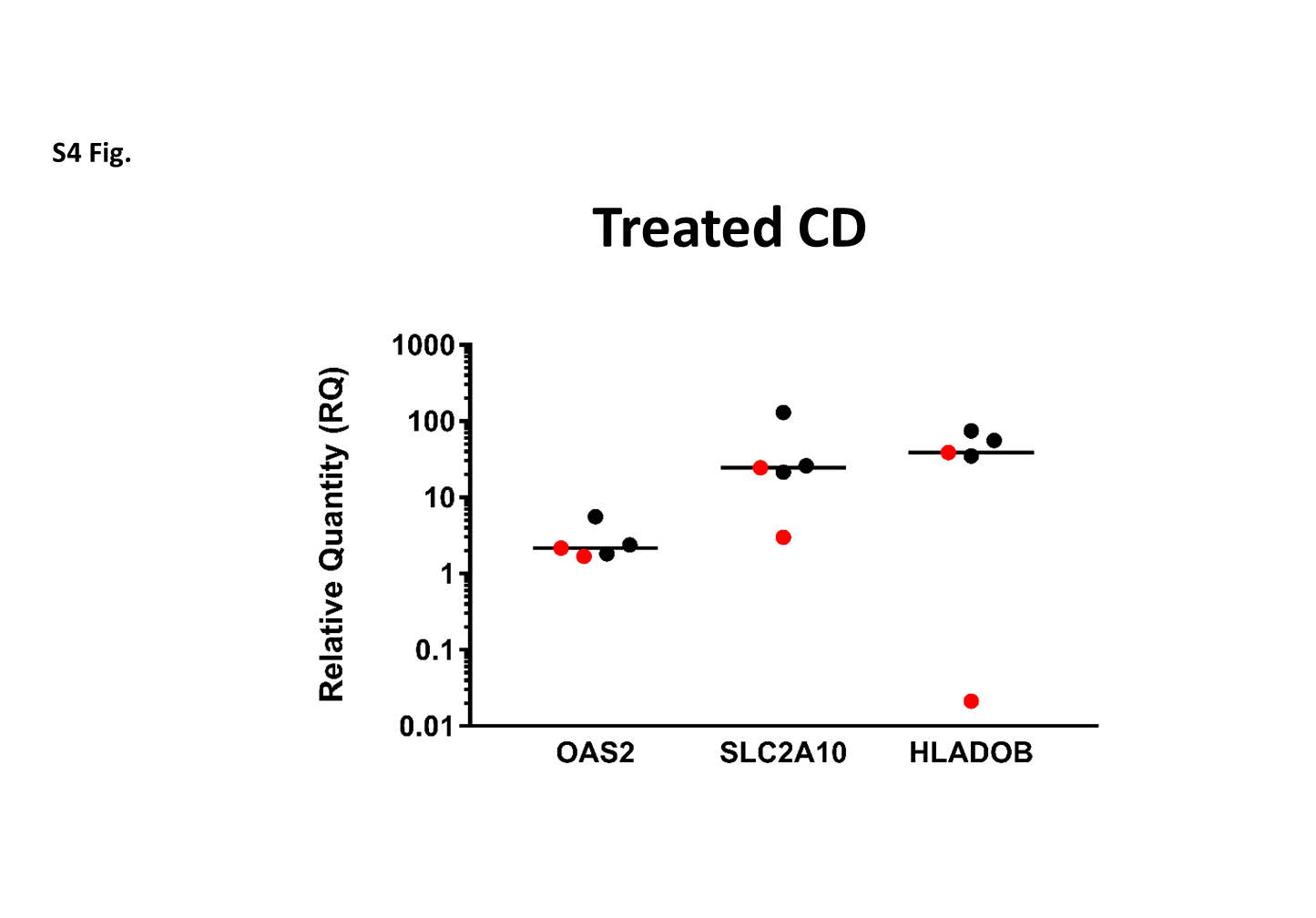

Supplement: S4 Fig — Expression levels, as estimated by qRT-PCR, of OAS2, SLC2A10 and HLA-DOB in IECs of CD patients on a gluten-free diet. Each dot represents IECs of one individual. Black dots represents IECs of patients with serum concentration of anti-tTG2 IgA ≤ 6 U/mL and pathological score Marsh 0 and red dots represents IECs of patients with anti-tTG2 IgA concentrations > 6 U/mL and pathological score Marsh 0/1 or 1. For clinical details see S1 Table. For methods used see legend to Fig 1 and Material and methods. (TIF) [file pone.0185025.s004.tif]
