# Supplementary material for: Immunopathology of childhood celiac disease—Key role of intestinal epithelial cells
Source: PLoS One. 2017 Sep 21;12(9):e0185025. doi: 10.1371/journal.pone.0185025 (PMC5608296; doi:10.1371/journal.pone.0185025)
Supplement: S1 Table — (DOCX) [file pone.0185025.s005.docx]

**S1 Table. Description of study subjects.**

| **Patient code** | **Age at biopsy (Years)** | **Sex (F/M)** | **Serum anti-tTG2 IgA (U/mL)** | **Pathology score (Marsh)** | **Type of analysis performed** |
| --- | --- | --- | --- | --- | --- |
| **IECs of active CD** | | | | | |
| 239 | 13.8 | F | 30° | 3a | 1^#^ |
| 244 | 13.1 | F | >100 | 3b | 1, 2, 3 |
| 245 | 12.9 | F | >100 | 3c | 1, 2, 3 |
| 246 | 12.7 | F | 5.7 | 3a | 1, 2, 3 |
| 251 | 13.0 | M | >100 | 3a | 2, 3 |
| 257 | 13.2 | F | >100 | 3a | 4 |
| 263 | 13.0 | F | >100 | 3c | 3 |
| 264 | 3.2 | F | 17 | 3a | 3 |
| 296 | 2.1 | M | >100 | 3b | 4 |
| 317 | 4.5 | M | 70 | 3b | 3 |
| 322 | 9.2 | M | 39 | 3a | 2, 3 |
| 323 | 9.2 | F | 28 | 3c | 2, 3 |
| 341 | 12.4 | F | >100 | 3c | 3 |
| 343 | 15.5 | M | 13 | 3a | 4 |
| 358 | 8.6 | F | 17 | 3a | 3 |
| 368 | 6.6 | F | >100 | 3a | 3 |
| 371 | 9.8 | F | 78 | 3c | 4, 5 |
| 381 | 12.3 | F | 77 | 3c | 4, 5 |
| 390 | 16.4 | F | >100 | 3c | 4, 5 |
| 474 | 7.2 | M | 49 | 3c | 3 |
| 477 | 3.3 | F | 21 | 3a | 3 |
| **IECs of treated CD** | | | | | |
| 340 (251)* | 14.3 | M | 18 | 1 | 2, 3 |
| 344 (246) | 14.1 | F | 1.6 | 0 | 2, 3 |

**S1 Table. Continued 1**

| **Patient code** | **Age at biopsy (Years)** | **Sex (F/M)** | **Serum anti-tTG2 IgA (U/mL)** | **Histology score (Marsh)** | **Type of analysis performed** |
| --- | --- | --- | --- | --- | --- |
| **IECs of treated CD** | | | | | |
| 346 | 14.6 | F | 3.7 | 0 | 2, 3 |
| 348 | 14.5 | F | 5.4 | 0 | 2, 3 |
| 349 (244) | 14.6 | F | 8.4 | 0/1 | 2, 3 |
| **IECs of Clinical Controls** | | | | | |
| 234 | 15.8 | F | <1 | 0 | 1 |
| 235 | 15.8 | F | <1 | 0 | 1, 3 |
| 236 | 5.1 | M | <1 | 0 | 1, 3 |
| 247 | 5.2 | F | ND | 0 | 1, 2 |
| 267 | 15.6 | M | <1 | 0 | 2, 3 |
| 269 | 7.7 | F | <1 | 0 | 2, 3 |
| 279 | 16.6 | F | <1 | 0 | 4 |
| 280 | 1.4 | F | <1 | 0 | 4 |
| 324 | 4.5 | F | <1 | 0 | 2, 3 |
| 357 | 6.3 | F | <1 | 0 | 2, 3 |
| 373 | 10.0 | F | <1 | 0 | 4, 5 |
| 377 | 17.0 | M | <1 | 0 | 4, 5 |
| 383 | 15.0 | F | <1 | 0 | 4, 5 |
| 473 | 1.4 | F | <1 | 0 | 3 |
| 476 | 10.2 | M | <1 | 0 | 3 |
| 478 | 9.7 | M | <1 | 0 | 3 |
| 537 | 16.9 | M | <1 | 0 | 6 |
| 547 | 15.1 | M | <1 | 0 | 6 |
| 552 | 17.8 | M | <1 | 0 | 6 |
| 566 | 1.1 | F | <1 | 0 | 6 |

**S1 Table. Continued 2**

| **Patient code** | **Age at biopsy (Years)** | **Sex (F/M)** | **Serum anti-tTG IgA (U/mL)** | **Histology (Marsh score)** | **Type of analysis performed** |
| --- | --- | --- | --- | --- | --- |
| **IECs of Clinical Controls** | | | | | |
| 567 | 16.6 | F | <1 | 0 | 6 |
| 572 | 17.7 | F | <1 | 0 | 6 |
| **CD3^+^ IELs of active CD** | | | | | |
| 221 | 12.8 | F | >100 | 3c | 1 |
| 289 | 10.7 | F | 69 | 3c | 1 |
| 290 | 10.7 | M | >100 | 3c | 1 |
| 375 | 12.8 | F | >100 | 3a | 1 |
| 378 | 4.1 | F | >100 | 3a | 1 |
| **CD3^+^ IELs of Clinical Controls** | | | | | |
| 250 | 16.3 | F | 1.6 | 0 | 1 |
| 271 | 8.6 | F | 1.1 | 0 | 1 |
| 272 | 3.2 | M | <1 | 0 | 1 |

F, female; M, male; ND, not done

°, Serum concentrations of anti-tTG2 IgA were determined using ImmunoCap 250 fluoroenzyme immunoassay, ThermoFisher Diagnostics, Uppsala, Sweden

*, Number within parentheses indicates the patient code at first biopsy

^#^, *Type of analysis performed*:

1 = Genome-wide hybridization bead array screening for gene expression using biotinylated double stranded cRNA library

2 = Quantitative real-time PCR-array using cDNA library

3 = Quantitative real-time RT-PCR using total RNA of individual samples

4 = Immunohistochemistry on frozen, paraformaldehyde fixed tissue sections

5 = Immunofluorescence on frozen, paraformaldehyde fixed tissue sections

6 = Enteroid culture
